# Impact of Auxiliary Task Heterogeneity on Dose Prediction: A Controlled Study of Optimization Coupling and Dose Outcomes

**DOI:** 10.1002/acm2.70906

**Published:** 2026-11-09

**Authors:** Yusi Xie, Haiming Yang, Chengxian Ma, Wenjie Liang, Li Mo, Liuke Liang, Qingguo Fu

**Affiliations:** ^1^ Department of Radiotherapy Technology Center Guangxi Medical University Cancer Hospital Nanning Guangxi China; ^2^ Department of Radiation Oncology Guangxi Medical University Cancer Hospital Nanning Guangxi China

**Keywords:** auxiliary supervision, breast cancer, deep learning, dose prediction, IMRT, radiotherapy

## Abstract

**Background:**

Auxiliary‐task training has been increasingly explored in radiotherapy dose prediction to incorporate planning‐relevant priors, such as patient anatomy, beam geometry, and spatial information, into model optimization. However, when auxiliary tasks differ in their physical meaning, input representation, and prediction target, their roles cannot be adequately understood from final prediction performance alone. Particularly in auxiliary‐supervision frameworks without shared feature representations, it remains unclear whether an auxiliary task establishes an effective optimization connection with the primary network, actually uses the additional information provided to it, and produces stable performance effects.

**Purpose:**

The purpose of this study was to compare heterogeneous auxiliary tasks for left‐sided breast IMRT dose prediction under controlled conditions and to evaluate their relationships with the primary dose‐prediction task in terms of optimization coupling, information use, and performance robustness.

**Materials and Methods:**

We implemented an independently parameterized auxiliary‐supervised joint optimization framework comprising a primary 3D dose prediction network and three auxiliary networks targeting patient anatomy, beam geometry, and isocenter‐related spatial information, without shared encoders or intermediate features. Specifically, we evaluated a dose‐only baseline (DS) and four auxiliary‐supervision configurations combining dose prediction with beam‐geometry (BS), center‐related (CS), patient‐structure (PS), or all auxiliary supervision (AS). Using data from 30 patients, we generated 130 plans per patient via bounded perturbations of beam angles and isocenter position, and evaluated all configurations on 650 test plans from held‐out patients under identical training settings. Prediction performance was first assessed using voxel‐wise errors and dosimetric endpoints. Gradient analysis was then used to quantify direct optimization coupling between auxiliary losses and DoseNet, while a matched CT + predicted‐dose versus CT‐only input ablation evaluated the use of predicted‐dose information by the Beam and Center tasks. Finally, performance robustness was assessed across multiple random seeds and under composite‐loss and MSE‐only primary dose objectives.

**Results:**

DS achieved the lowest overall MAE/RMSE, while BS showed comparable performance: under the composite‐loss setting, BS exhibited more consistent cross‐seed improvements across several clinically relevant dosimetric endpoints. Further analyses revealed distinct pathways among the auxiliary tasks. Both Beam and Center were directly connected to DoseNet through the predicted dose, but Beam showed substantially larger relative gradient magnitudes, and inclusion of the predicted dose improved Beam prediction across all three random seeds. In contrast, Center showed no consistent benefit from predicted‐dose information, while Patient had no direct auxiliary‐gradient pathway to DoseNet. Changing the primary dose objective from the composite loss to MSE‐only substantially altered the endpoint‐level effects and cross‐seed consistency of the auxiliary configurations, with the most pronounced loss of consistency observed for BS.

**Conclusion:**

The role of auxiliary supervision cannot be determined from final performance changes alone. Optimization coupling, information use, and performance robustness represent distinct dimensions that should be evaluated separately. Gradient analysis, input ablation, performance comparisons across random seeds and primary loss formulations together provide a more complete framework for evaluating auxiliary supervision than performance comparison alone.

## 1 INTRODUCTION

Radiotherapy dose prediction aims to provide fast, voxel‐wise estimates of three‐dimensional dose distributions that can support treatment planning quality assessment and optimization.^1^, ^2^, ^3^ Within the development of deep learning, three‐dimensional convolutional networks, including U‐Net‐based architectures and attention mechanisms, have been widely used for direct volumetric dose prediction.^4^, ^5^, ^6^, ^7^ Beyond architectural design, an important question is how planning‐relevant priors, such as patient anatomy, beam geometry, and spatial information, can be effectively incorporated into the learning process.^2^, ^8^, ^9^, ^10^

A common strategy is to provide these planning priors as additional input channels to the dose‐prediction network.^8^, ^9^, ^10^ However, the presence of an input does not establish whether the model actually uses that information, nor whether a related prediction objective imposes an additional optimization constraint on the primary dose network. An alternative is therefore to formulate planning priors as auxiliary prediction tasks and optimize them jointly with the primary dose‐prediction objective.^11^, ^12^, ^13^, ^14^

Such designs are often discussed within the broader context of multi‐task learning (MTL). Conventional MTL frameworks typically involve shared encoders, shared intermediate representations, or other forms of parameter sharing through which multiple tasks jointly shape a common feature representation.^15^ Prior MTL dose prediction studies have adopted shared representations in different ways. For example, Tan et al. and Wen et al. incorporated dose‐related auxiliary objectives such as isodose‐line and dose‐gradient prediction within shared multi‐task architectures,^11^, ^13^ whereas Maniscalco et al. developed a multi‐task model that simultaneously predicted dose distributions for multiple radiotherapy modalities using a shared encoder–decoder backbone with task‐specific output layers.^16^ In contrast, the present study uses independently parameterized networks without shared feature representations and compares heterogeneous auxiliary tasks that differ in physical meaning, input conditioning, and prediction target.

However, when different tasks are implemented using independently parameterized networks without shared feature representations or parameter‐sharing constraints, the interaction mechanism differs from conventional hard‐ or soft‐parameter‐sharing MTL.^15^ In the present study, we therefore describe this design as auxiliary‐supervised joint optimization, consistent with the broader auxiliary‐learning paradigm in which auxiliary objectives are optimized together with a primary task to support its learning.^17^

Within such a framework, the role of an auxiliary task cannot be adequately characterized by final prediction performance alone. For the purposes of this study, we distinguish three complementary aspects of auxiliary supervision: optimization coupling, referring to how an auxiliary objective contributes to the optimization of the primary network^18^; information use, referring to whether the auxiliary task actually uses the additional information provided by its design^19^; and performance robustness, referring to whether the resulting performance effect remains consistent across random seeds and training objectives.^20^ These dimensions describe different properties of auxiliary supervision and therefore require separate evaluation.

In this study, left‐sided breast postoperative intensity‐modulated radiotherapy (IMRT) dose prediction was used as a controlled experimental setting, where target and organ‐at‐risk anatomy, beam arrangement, and isocenter position have clear spatial relationships with the resulting dose distribution.^21^, ^22^, ^23^ We constructed an independently parameterized auxiliary‐supervision framework comprising a primary dose‐prediction network and three auxiliary networks targeting patient anatomy (Patient), beam geometry (Beam), and isocenter‐related spatial information (Center), without shared encoders or intermediate features. Beam and Center receive CT together with the current predicted dose and therefore form direct computational pathways to the primary dose network through the predicted dose, whereas Patient receives CT alone and does not form such a direct auxiliary‐gradient pathway.

Under matched data splits and training conditions, we compare five task configurations: dose only prediction (DS), dose + beam supervision (BS), dose + center supervision (CS), dose + patient supervision (PS), and an all‐task configuration (AS). Prediction performance was first evaluated using voxel‐wise errors and dosimetric endpoints. Gradient analysis was then used to assess direct optimization coupling between auxiliary objectives and DoseNet; a matched CT + predicted‐dose versus CT‐only ablation was used to evaluate the use of predicted‐dose information; and multiple random seeds together with composite‐loss and MSE‐only settings were used to assess the robustness of auxiliary‐task effects. Through these analyses, this study aims to evaluate auxiliary supervision along three dimensions—optimization coupling, information use, and performance robustness—and to provide a practical validation strategy for auxiliary‐task selection, mechanism verification, and robustness assessment in future radiotherapy dose‐prediction frameworks.

## 2 METHODS

### 2.1 Patient Cohort, Ethics, and Plan Generation

This retrospective study included 30 patients with left‐sided breast cancer treated with postoperative IMRT under a conventionally fractionated prescription of 50 Gy in 25 fractions. All cases were collected from a single tertiary cancer center between May 2019 and January 2023. Eligible patients had a single‐isocenter treatment setup, complete planning CT images, and complete structure delineations covering the planning target volume (PTV), contralateral breast, heart, bilateral lungs, spinal cord, and external body contour. Target volumes and organs at risk (OARs) were delineated in routine clinical practice by radiation oncologists. Additional cohort characteristics are reported in Supplement A.

The study was approved by the institutional ethics committee (Approval No. KY2023625). All data were de‐identified prior to analysis. Given the retrospective nature of the study and the exclusive use of anonymized imaging and dosimetric information, the requirement for informed consent was waived (Supplement A).

To support a controlled comparison of heterogeneous auxiliary‐supervision configurations, reference dose distributions were generated using a unified automated planning pipeline implemented in the open‐source matRad framework (version 2.10.0). Optimization objectives were kept consistent across plans and followed planning goals for left‐sided breast irradiation. Detailed planning settings are provided in Supplement B.

For each patient, controlled variations in beam geometry and isocenter position were introduced. Starting from a reference six‐field IMRT arrangement, beam angles were perturbed within ±10° to generate 13 beam‐angle configurations. Ten isocenters were then combined with each beam‐angle configuration, yielding 130 plan parameter sets per patient (13 × 10) and 3900 candidate plans across the 30 patients. After integrity screening, 3863 plans were retained for analysis. To avoid information leakage between plan variants derived from the same anatomy, the dataset was split at the patient level into 22 patients for training (2,831 plans), three for validation (382 plans), and five for testing (650 plans). Descriptive patient‐level comparisons of anatomical volumes and reference dosimetric characteristics across the training, validation, and test splits are provided in Supplementary Tables S6 and S7, respectively. The clinical dose constraints and corresponding dose–volume objectives implemented for plan generation are summarized in Tables S1 and S2, respectively, with additional planning details provided in Supplement B.

### 2.2 Preprocessing

All CT images, structure masks, and reference dose distributions were resampled to a common 128 × 128 × 128 voxel grid with an isotropic spacing of 4 × 4 × 4 mm^3^. CT intensities were clipped to a fixed Hounsfield Unit (HU) range and min–max normalized to [0, 1]. Light smoothing was applied to selected geometric input channels including structure boundaries and beam‐related representations. All preprocessing procedures were kept identical across dataset splits and task configurations. Detailed interpolation, normalization, and smoothing parameters are provided in Supplement C.

### 2.3 Task Design and Auxiliary Supervision Framework

Following preprocessing, the proposed framework comprised one primary dose‐prediction task and three auxiliary tasks related to patient anatomy, beam geometry, and isocenter‐related spatial information. Each task was implemented using an independently parameterized network, with no shared encoder or intermediate feature representation across branches. Task interaction was introduced through the joint training objective rather than through shared features. An overview of the training workflow is summarized in Figure 1.

**FIGURE 1:**
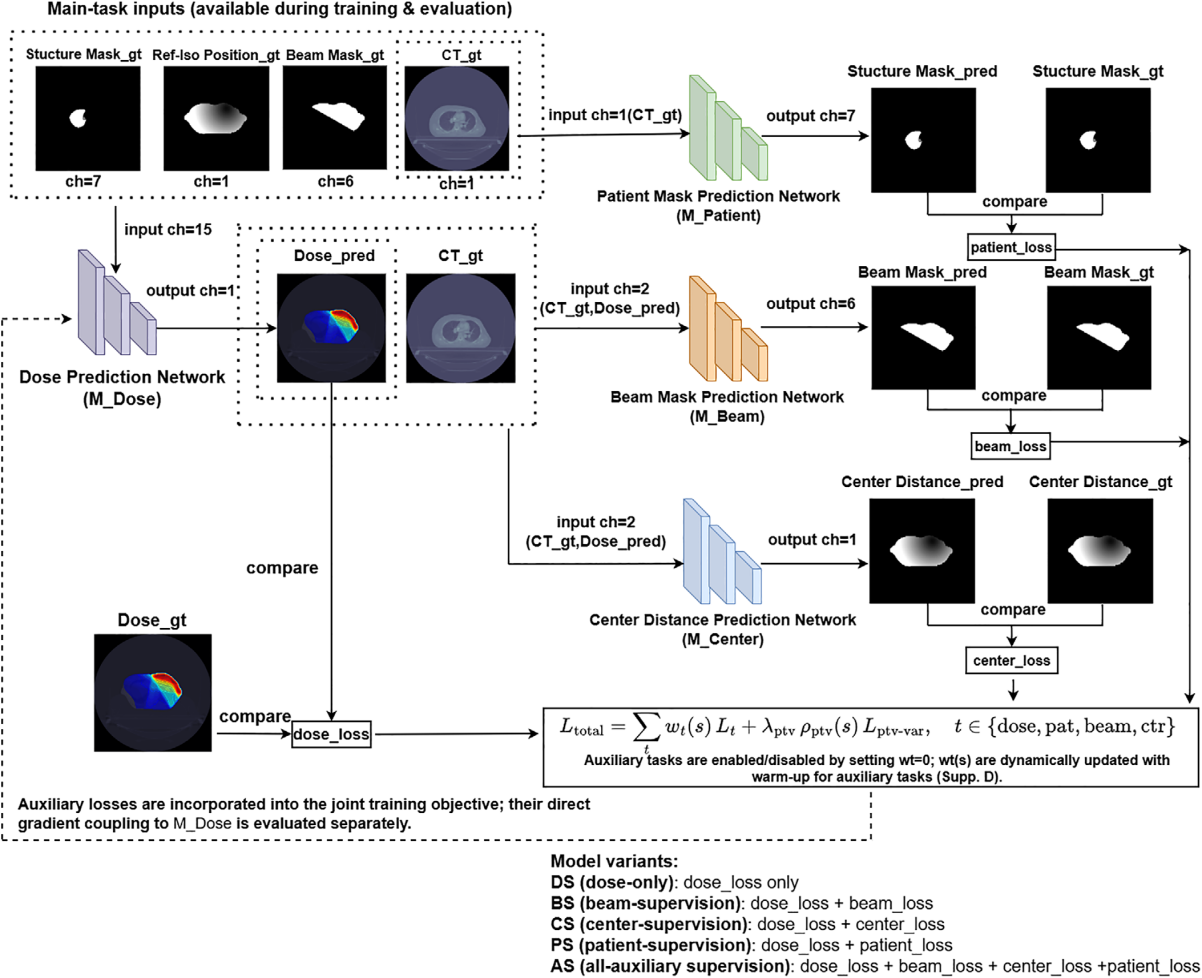
Auxiliary‐supervision training workflow and task‐configuration design. Schematic overview of the joint training framework used in this study. The main dose prediction network (M_Dose) takes the same set of inputs during training, evaluation, and inference to generate the predicted dose distribution. Auxiliary task networks are introduced only during training and evaluation to provide additional supervision signals and are not involved during inference. The overall training objective is formulated as a weighted sum of the dose prediction loss and auxiliary task losses, such that auxiliary objectives influence the optimization of M_Dose through loss‐level coupling rather than shared feature representations. The Beam and Center auxiliary networks receive the predicted dose together with CT input, whereas the Patient network receives CT alone. Corresponding auxiliary losses are computed against task‐specific ground truth. Different task configurations (DS, BS, CS, PS, AS) are realized by enabling or disabling specific auxiliary loss terms while keeping the network architectures, input data, and training settings fixed. Auxiliary losses are incorporated using a predefined weighting and warm‐up strategy to stabilize joint optimization. This figure illustrates the conceptual training logic and interactions between loss terms, rather than implementation‐level details.

The primary dose‐prediction task was performed by M_Dose (hereafter referred to as DoseNet), whereas M_Patient, M_Beam, and M_Center provided patient‐structure, beam‐geometry, and isocenter‐related auxiliary supervision, respectively. The auxiliary tasks were designed to represent distinct anatomical and geometric aspects of treatment planning. Their task‐specific inputs and outputs are described in Section 2.4.

Based on these task definitions, five training configurations were evaluated: dose only (DS), dose + beam supervision (BS), dose + center supervision (CS), dose + patient‐structure supervision (PS), and all auxiliary tasks (AS). Task configurations differed only in the auxiliary loss terms enabled during joint optimization; the dose‐network input representation, data split, and overall training procedure were otherwise kept consistent across configurations.

### 2.4 Network Architecture and Implementation

The main dose predictor and the three auxiliary predictors were implemented using task‐specific 3D U‐Net–based architectures. Each network was instantiated with its own parameters, and no feature sharing was used between branches. An overview of the network architecture is provided in Figure 2 and detailed layer configurations are provided in Figure S1.

**FIGURE 2:**
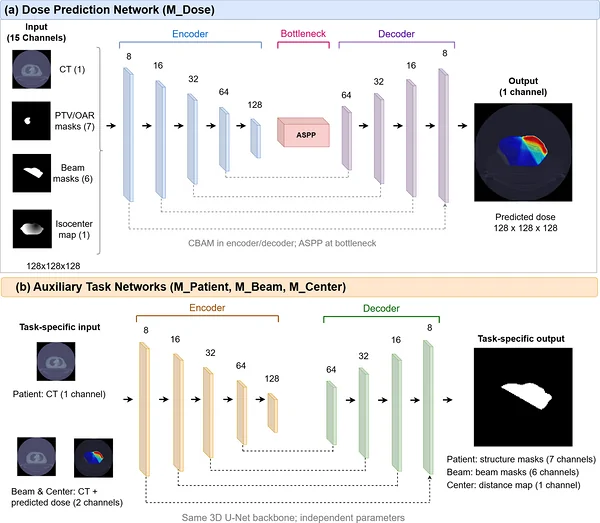
Overview of the dose‐prediction and auxiliary‐task network architectures. (a) Five‐level 3D U‐Net–based dose‐prediction network M_Dose with CBAM modules in the encoder/decoder and ASPP at the bottleneck. (b) Independently parameterized auxiliary networks for patient structures, beam geometry, and isocenter‐related spatial information. Task‐specific input and output channels are indicated in the figure. Detailed layer‐level configurations are provided in Supplementary Figure S1. Abbreviations: ASPP, atrous spatial pyramid pooling; CBAM, convolutional block attention module; OAR, organ at risk; PTV, planning target volume.

#### 2.4.1 Main Dose Prediction Network

The main network, M_Dose, predicts a continuous three‐dimensional dose distribution $\hat{D}\in R^{128\times 128\times 128}$, with non‐negativity enforced using a Softplus activation at the output layer. M_Dose receives a 15‐channel input comprising one CT channel, seven anatomical structure‐mask channels (PTV, contralateral breast, heart, left lung, right lung, spinal cord, and body), six beam‐geometry mask channels corresponding to the six treatment fields, and one isocenter‐related spatial encoding channel (1 + 7 + 6 + 1 = 15 channels).

The backbone of M_Dose follows a five‐level encoder–decoder architecture with skip connections. To support volumetric context aggregation, attention modules (CBAM) and a multi‐scale atrous spatial pyramid pooling (ASPP) block are incorporated. These components are used consistently across all experiments.

#### 2.4.2 Auxiliary Networks

The auxiliary networks are based on a U‐Net–style encoder–decoder backbone, but use simplified variants of the main dose network architecture without CBAM modules, ASPP blocks, or residual enhancements. Although they follow the same general architectural template, M_Beam, M_Center and M_Patient are instantiated independently and do not share parameters.

The patient structure network, M_Patient, receives a single‐channel CT volume as input and predicts seven anatomical structure‐mask channels corresponding to the PTV, contralateral breast, heart, left lung, right lung, spinal cord, and body.

The beam‐geometry network, M_Beam, and the isocenter‐related network, M_Center, each receive a two‐channel input formed by concatenating the single‐channel CT volume with the single‐channel dose prediction $\hat{D}$ produced by M_Dose. M_Beam predicts six beam‐mask channels corresponding to the six treatment fields, whereas M_Center predicts a single‐channel isocenter‐related spatial representation.

### 2.5 Training Objective and Task Weighting

The training objective was defined as a weighted combination of the main dose prediction loss and auxiliary losses enabled for each task configuration:

(1) $$L=w_{dose}\;L_{dose}+w_{pat}L_{pat}+w_{beam}L_{beam}+w_{ctr}L_{ctr}$$

where auxiliary loss terms corresponding to inactive tasks were set to zero. The active task weights were adjusted according to a predefined dynamic weighting schedule, including progressive introduction and normalization of the auxiliary losses. The same weighting strategy was applied across task configurations and random seeds. Baseline hyperparameter settings, grid‐search results, and the final effective hyperparameters are provided in Tables S3–S5, respectively, with full details of the weighting schedule provided in Supplement D.

The main dose loss, $L_{\mathrm{Dose}}$, was a predefined composite objective combining voxel‐wise, spatial, and ROI‐level dose constraints. The same composite dose objective was used for all five configurations in the primary experiments. The Beam and Patient tasks were optimized using segmentation‐based losses $L_{beam}$ and $L_{pat}$ combining Dice and cross‐entropy terms, whereas the Center task used a voxel‐wise regression loss $L_{ctr}$. Full definitions of the dose and auxiliary losses, including the PTV‐focused regularization term, are provided in Supplement D.

### 2.6 Training Procedure and Evaluation Protocol

#### 2.6.1 Training Procedure

All models were implemented in PyTorch and trained using the Adam optimizer with an initial learning rate of 2 × 10^−3^ and a batch size of 1. Early stopping was applied based on validation performance using the same criterion across task configurations. Each configuration was independently trained using three random seeds to assess the reproducibility of auxiliary‐task effects. Unless otherwise specified, predictions from the three seeds were averaged at the plan level prior to cohort‐level summarization. Detailed training hyperparameters are provided in Supplement D.

#### 2.6.2 Primary Evaluation

Performance was evaluated on 650 test plans from held‐out patients. Voxel‐wise dose accuracy was assessed using mean absolute error (MAE) and root mean squared error (RMSE) within the body region. Dosimetric performance was further evaluated using structure‐level dose–volume histogram (DVH) endpoints and target‐quality indices. Comparisons between auxiliary‐supervision configurations and the dose‐only baseline (DS) were performed on matched test plans. Additional spatial evaluation using Dice similarity coefficients (DSC) of isodose regions across multiple dose thresholds was conducted for the Body and PTV regions, with full results provided in Tables S13 and S14. Exploratory analyses further examined the plan‐level association between auxiliary‐task losses and dose‐prediction errors using Spearman rank correlations and quartile‐based stratification, as reported in Tables S15 and S16, respectively. Definitions of all evaluation metrics and details of the statistical analysis protocol are provided in Supplement G.

#### 2.6.3 Task‐Specific Gradient Analysis

To assess the direct optimization coupling between each auxiliary objective and the dose‐prediction network, we quantified the gradient norm of the weighted auxiliary loss with respect to the M_Dose parameters and normalized it by the corresponding dose‐loss gradient norm. Gradient contributions were sampled across training checkpoints for each random seed. Seed‐specific medians and interquartile ranges (IQRs) were calculated, and cross‐seed summaries were derived from the three seed‐specific median values. The presence of a direct gradient connection was additionally assessed by the number of sampled checkpoints with a non‐zero auxiliary gradient to M_Dose. The same analysis was performed under both the composite‐loss and MSE‐only settings.

#### 2.6.4 Dose‐Loss Sensitivity Analysis

To assess whether auxiliary‐task effects were robust to the primary dose objective, the original composite dose loss was replaced by voxel‐wise MSE, while the five task configurations, data splits, network architectures, and evaluation procedure were otherwise retained. Each configuration was retrained using the same three random seeds. For each selected dosimetric endpoint and seed, the paired effect relative to DS was defined as

$$\;\Delta_{s}=E_{\mathrm{aux},s}\;-E_{\mathrm{DS},s},$$

where E denotes the absolute endpoint error relative to the ground truth; therefore, $\Delta_{s}<0$ indicates lower error than DS. Cross‐seed consistency was summarized by the number of seeds showing improvement and compared between the composite‐loss and MSE‐only settings.

#### 2.6.5 Auxiliary‐Input Dependence Analysis

To assess whether the Beam and Center tasks used information from the predicted dose beyond CT alone, the corresponding pretrained DS model for each seed was frozen and used to generate dose predictions. Beam and Center networks were then retrained under two matched conditions: CT concatenated with the frozen predicted dose (CT+dose) and CT concatenated with a zero‐valued second channel (CT‐only). The two conditions used the same two‐channel architecture, matched initialization, data order, and training settings. Paired early stopping was applied within each task and seed, and the resulting models were evaluated on the same 650 held‐out test plans. The contribution of the predicted‐dose input was quantified as

$$\mathrm{Relative}\;\mathrm{loss}\;\mathrm{reduction}\;=\frac{L_{\mathrm{CT}-\mathrm{only}}-L_{\mathrm{CT}+\mathrm{dose}}}{L_{\mathrm{CT}-\mathrm{only}}}\;\times 100\%.$$

Positive values indicate lower auxiliary‐task loss when predicted‐dose information was included.

### 2.7 Computational Environment

The experiments in this study were conducted on a Windows 11 operating system with an AMD Ryzen 7 5800H processor (3.20 GHz) and 16.0 GB of RAM. The GPU used was an NVIDIA RTX 3070 with 8 GB of VRAM. The software environment included Python 3.8, CUDA 11.0, and PyTorch 1.12.0.

### 2.8 AI Tool Usage Statement

During the preparation of this work, the authors used ChatGPT‐5.2 to enhance the language and readability of the manuscript. After utilizing this tool, the authors reviewed and edited the content as necessary and take full responsibility for the content of the published article.

## 3 RESULTS

Unless otherwise specified, reported performance metrics are based on plan‐level metrics averaged across three random seeds, following the evaluation protocol described in Section 2.6. Seed‐specific results are additionally reported for analyses of cross‐seed robustness, optimization coupling, and predicted‐dose dependence. Further details of the evaluation and statistical procedures are provided in Supplement G.

### 3.1 Global Voxel‐Wise Performance

As summarized in Table 1, DS achieved the lowest overall mean MAE (2.95 ± 0.63 Gy) and RMSE (5.19 ± 1.19 Gy) across the test set. BS showed very similar values (MAE: 2.96 ± 0.78 Gy; RMSE: 5.19 ± 1.37 Gy). CS, PS, and AS showed slightly higher mean MAE and RMSE than DS. Formal paired comparisons (including effect sizes and confidence intervals) are reported for completeness in Supplement E (Table S8). Seed‐specific MAE and RMSE results for each model configuration under the composite‐loss setting are provided in Supplement E (Table S17). Additional qualitative examples of predicted dose distributions, voxel‐wise error patterns, and DVH agreement for representative test cases are provided in Supplementary F (Figures S3–S5).

**TABLE 1:** Overall voxel‐wise prediction errors

Model	MAE(Gy) mean ± SD	RMSE(Gy) mean ± SD
DS	**2.95 ± 0.63**	**5.19 ± 1.19**
BS	2.96 ± 0.78	5.19 ± 1.37
CS	3.01 ± 0.59	5.31 ± 1.11
PS	3.05 ± 0.54	5.36 ± 1.04
AS	3.03 ± 0.64	5.30 ± 1.16

Note: Mean ± SD of MAE and RMSE across 650 test plans for the five task configurations, averaged across three independent random seeds. Values are rounded to two decimals, and the lowest mean value for each metric is highlighted in bold. Abbreviations: DS, dose‐only; BS, dose + beam supervision; CS, dose + center supervision; PS, dose + patient‐structure supervision; AS, all auxiliary tasks.

### 3.2 Endpoint‐Level Effects and Robustness Across Loss Formulations

Prediction bias was defined as Prediction − Ground Truth for selected target and OAR dose–volume endpoints. Table 2 summarizes the mean bias across the five task configurations under the composite‐loss setting. Negative values indicate underestimation relative to the reference.

**TABLE 2:** Key structure‐level bias in clinically relevant endpoints (Prediction − GT)

Metric	GT mean	DS bias	BS bias	CS bias	PS bias	AS bias
PTV D95(Gy)	45.35	−1.73	−**0.67**	−1.89	−2.38	−1.63
PTV Dmean(Gy)	45.77	**0.04**	1.21	0.30	−0.54	0.15
Heart Dmean(Gy)	9.21	−1.73	−**0.95**	−1.40	−1.64	−1.25
Cord Max(Gy)	26.30	−6.70	−**5.01**	−6.28	−7.04	−5.82
LungL Dmean(Gy)	16.94	−2.69	−**1.68**	−2.29	−2.89	−2.22
LungL V20(fraction)	0.37	−0.06	−**0.03**	−0.05	−0.06	−0.04
Body Dmean(Gy)	8.80	−0.58	−**0.22**	−0.55	−0.75	−0.46

Note: Biases are reported for selected PTV and OAR metrics that are emphasized in the main text. Across the 650 test plans for five task configurations, results are averaged across three independent random seeds. Negative values indicate underestimation. Values are rounded to two decimals; best performance is determined using full‐precision values. For each metric, the bias value with the smallest absolute magnitude is highlighted in bold. Abbreviations: GT, ground truth; DS, dose‐only baseline; BS, dose + beam auxiliary task; CS, dose + center auxiliary task; PS, dose + patient‐structure auxiliary task; AS, all auxiliary tasks; CI, conformity index; Dmean, mean dose; LungL, ipsilateral (left) lung; PTV, planning target volume; Vx, volume fraction receiving at least x Gy (e.g., V5, V20).

For target‐related endpoints, BS showed a smaller absolute bias than DS for PTV D95, whereas DS showed the smallest absolute bias for PTV Dmean. For OAR‐related endpoints, BS showed the smallest absolute bias for Heart Dmean, Cord max, LungL Dmean, LungL V20, and Body Dmean among the reported configurations. Complete endpoint results are provided in Supplement E (Tables S9–S12, Figure S2).

The consistency of these endpoint‐level effects across random seeds is summarized in Figure 3. Under the composite‐loss setting, BS showed lower absolute GT error than DS in all three seeds for PTV D95, PTV mean dose, Cord Dmax, and LungL V20, and in two of three seeds for Heart Dmean. In contrast, AS, CS, and PS generally showed mixed seed‐wise directions across these endpoints.

**FIGURE 3:**
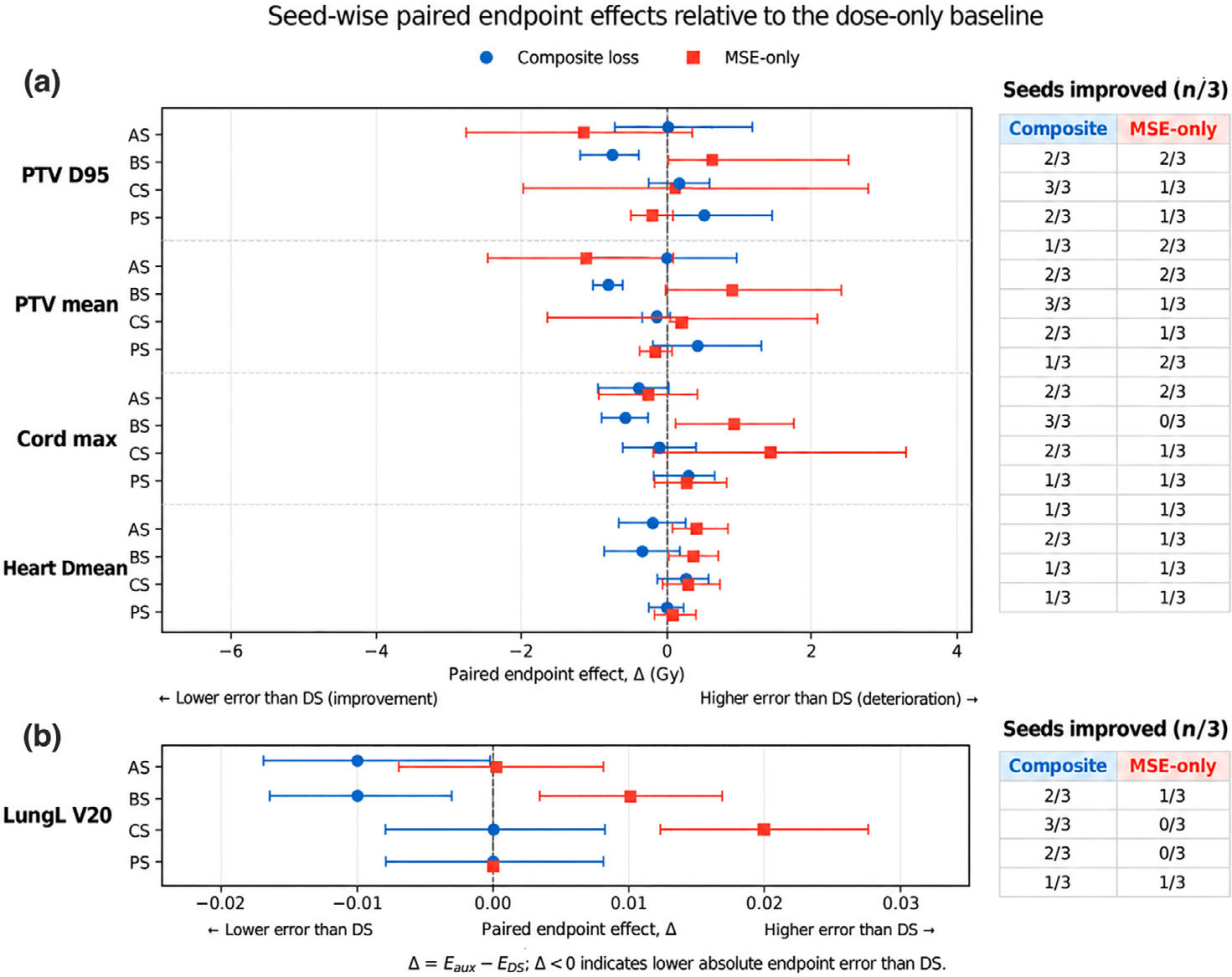
Seed‐wise paired endpoint effects relative to the dose‐only baseline under composite‐loss and MSE‐only training. For each endpoint and auxiliary‐supervision configuration, the paired effect was defined as the difference in absolute endpoint error relative to the corresponding DS model. Negative values indicate lower absolute GT error than DS, whereas positive values indicate higher error. Points and horizontal error bars represent the cross‐seed mean and standard deviation, respectively, across three random seeds. The right‐hand summaries indicate the number of seeds showing lower endpoint error than DS. (a) Dose‐based endpoints expressed in Gy, including PTV D95, PTV mean dose, spinal cord maximum dose, and heart mean dose. (b) Left‐lung V20. Blue circles represent the composite‐loss setting, and red squares represent MSE‐only training. Abbreviations: DS, dose‐only; AS, all auxiliary tasks; BS, beam supervision; CS, center supervision; PS, patient‐structure supervision; PTV, planning target volume.

When the primary dose objective was replaced by voxel‐wise MSE, the seed‐wise pattern changed substantially (Figure 3). For BS, the number of seeds showing lower absolute GT error than DS decreased from 3/3 to 1/3 for PTV D95 and PTV mean dose, from 3/3 to 0/3 for Cord Dmax and LungL V20, and from 2/3 to 1/3 for Heart Dmean. PS showed more limited changes: improvements increased from 1/3 to 2/3 seeds for PTV D95 and PTV mean dose, whereas Cord Dmax, Heart Dmean, and LungL V20 remained improved in only 1/3 seeds. AS showed comparatively limited changes in seed‐wise consistency, whereas CS generally exhibited reduced consistency for several endpoints. These results indicate that the direction and consistency of auxiliary‐task effects depended on the formulation of the primary dose objective. Detailed seed‐specific and cross‐loss results are provided in Supplement E (Tables S17–S21).

### 3.3 Task‐Specific Optimization Coupling Across Loss Formulations

To further characterize whether the task‐dependent performance patterns were accompanied by differences in direct optimization coupling, we quantified the relative gradient contribution of each auxiliary objective to the dose‐prediction network (Figure 4). Under the composite‐loss setting, the Beam objective consistently exhibited substantially larger relative gradient contributions than the Center objective across all three random seeds, whereas the Patient objective showed no direct gradient contribution to DoseNet.

**FIGURE 4:**
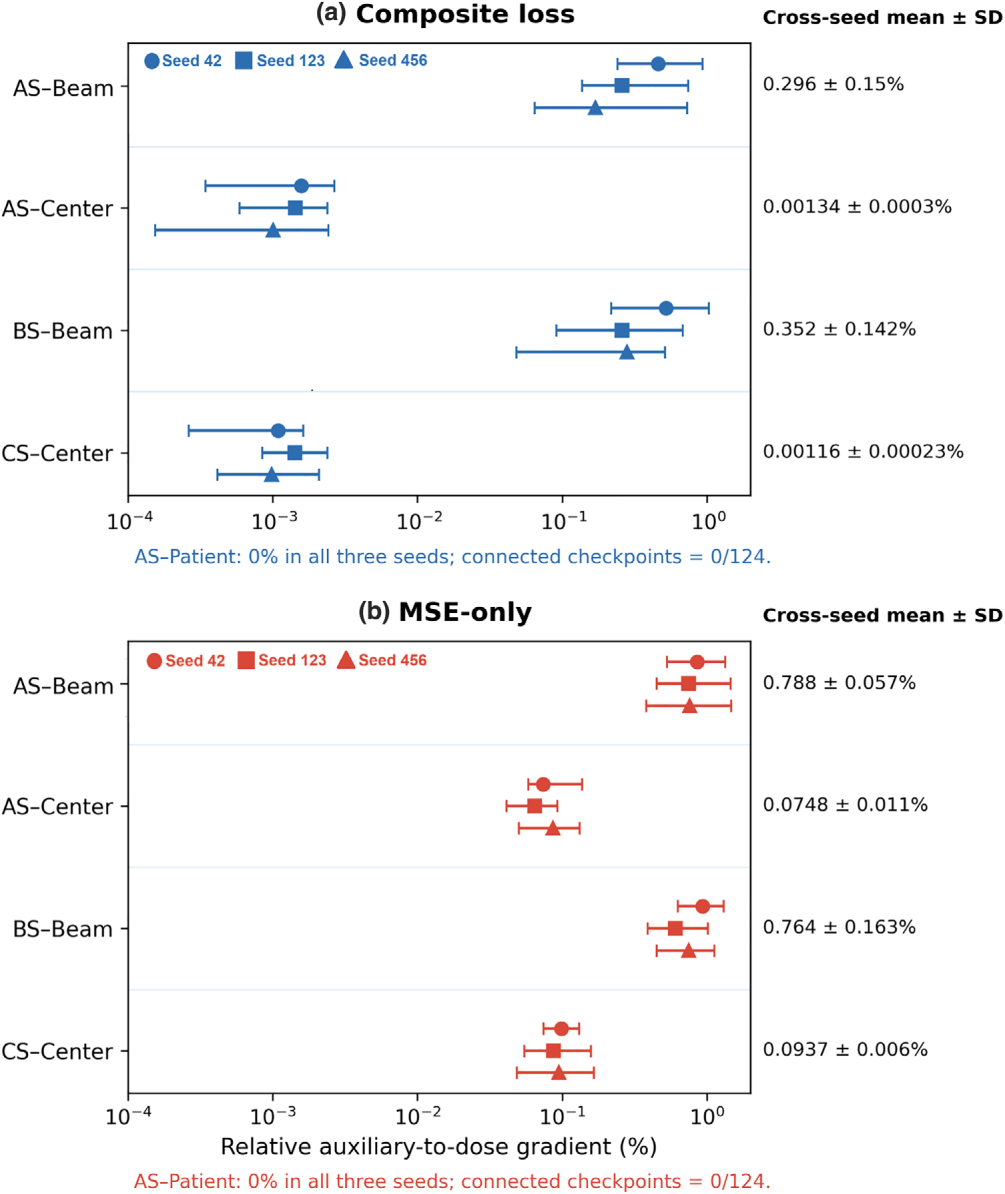
Task‐specific relative gradient contributions to the dose‐prediction network under composite‐loss and MSE‐only training. Relative gradient contribution was defined as the gradient norm of the weighted auxiliary loss with respect to the DoseNet parameters, normalized by the corresponding dose‐loss gradient norm. (a) Composite‐loss setting. (b) MSE‐only setting. Circles, squares, and triangles denote random seeds 42, 123, and 456, respectively. For each seed, points represent the median relative gradient contribution and horizontal error bars represent the interquartile range (IQR) across sampled checkpoints. Cross‐seed mean ± SD values, calculated from the three seed‐specific medians, are shown on the right. Because zero values cannot be displayed on the logarithmic axis, the Patient task is shown separately below each panel. Its relative gradient contribution was 0% for all three seeds, with 0/124 sampled checkpoints showing a non‐zero gradient connection. Beam and Center showed non‐zero gradient connections at all 124 sampled checkpoints for each seed.

This task‐specific hierarchy was preserved under MSE‐only training. Numerically larger relative gradient ratios were observed for both Beam and Center under MSE‐only training; however, the task‐specific ordering was preserved, with Beam remaining markedly larger than Center. The Patient objective again showed a relative gradient contribution of 0% across all three seeds. Across both loss formulations, non‐zero auxiliary gradients were detected at all sampled checkpoints for the Beam and Center objectives, whereas no non‐zero gradient connection was observed for the Patient objective.

These results demonstrate a consistent task‐specific ordering of direct optimization coupling across loss formulations: Beam showed the strongest coupling to DoseNet, Center showed a much weaker but non‐zero coupling, and Patient showed no direct auxiliary‐gradient pathway to the dose‐prediction network. Detailed seed‐specific gradient statistics are provided in Tables S22 and S23.

### 3.4 Predicted‐Dose Dependence of Geometry‐Related Auxiliary Tasks

Although both the Beam and Center auxiliary tasks receive the predicted dose as part of their input, the presence of this input pathway does not by itself establish that the auxiliary networks make meaningful use of the predicted‐dose information. To assess this, Beam and Center networks were evaluated under matched CT + predicted‐dose and CT‐only conditions (Table 3).

**TABLE 3:** Auxiliary‐task test losses with and without the predicted‐dose input across three random seeds

Task	Seed	Auxiliary loss, CT + predicted dose	Auxiliary loss, CT‐only	Relative improvement	Seeds with lower loss using dose
Beam	42	0.095 ± 0.008	0.143 ± 0.049	33.50%	3/3
	123	0.105 ± 0.023	0.151 ± 0.054	30.00%	
	456	0.190 ± 0.032	0.233 ± 0.050	18.10%	
Center	42	0.00675 ± 0.00349	0.00720 ± 0.00329	6.20%	1/3
	123	0.00744 ± 0.00409	0.00741 ± 0.00457	−0.40%	
	456	0.01113 ± 0.00647	0.00681 ± 0.00344	−63.40%	

Note: Values are auxiliary‐task losses evaluated on the 650 held‐out test plans and are reported as mean ± SD across test plans. For the CT + predicted dose condition, each auxiliary network received the CT volume concatenated with the dose predicted by the corresponding frozen dose‐only M_Dose model. For the CT‐only condition, the same two‐channel auxiliary‐network architecture was used, but the predicted‐dose channel was replaced by zeros. Beam and Center networks were retrained separately under the two matched input conditions. Relative loss reduction was calculated as $\left(L_{\mathrm{CT}-\mathrm{only}}-L_{\mathrm{CT}+\mathrm{dose}}\right)/L_{\mathrm{CT}-\mathrm{only}}\times 100\%$. Positive values indicate a lower auxiliary‐task loss when the predicted‐dose input was included, whereas negative values indicate a lower loss under the CT‐only condition. Beam and Center losses were calculated using their respective task‐specific loss functions and should not be compared directly in absolute magnitude.

For the Beam task, inclusion of the predicted‐dose input consistently reduced the auxiliary‐task loss across all three random seeds. The relative loss reductions were 33.5%, 30.0%, and 18.1% for seeds 42, 123, and 456, respectively, corresponding to lower loss with predicted‐dose information in 3/3 seeds.

In contrast, the Center task showed no consistent benefit from the predicted‐dose input across seeds. A 6.2% relative reduction in auxiliary loss was observed for seed 42, whereas relative changes of −0.4% and −63.4% were observed for seeds 123 and 456, respectively. Accordingly, lower auxiliary‐task loss with the predicted‐dose input was observed in only 1/3 seeds for the Center task.

These results indicate that the Beam task consistently benefited from access to the predicted‐dose information, whereas the Center task did not show stable dependence on this input across random seeds. This distinction complements the gradient‐based coupling analysis in Section 3.3 by separating the existence of a direct optimization pathway from the extent to which the auxiliary task actually uses the predicted‐dose information.

## 4 DISCUSSION

### 4.1 Auxiliary Supervision Does Not Consistently Improve Global Prediction Accuracy

In this study, we conducted a controlled comparison of heterogeneous auxiliary‐task designs for dose prediction in left‐sided breast IMRT. Using a unified plan‐generation pipeline with bounded beam‐angle and isocenter perturbations, we evaluated five task configurations under matched inputs and training settings: DS and four auxiliary‐supervision variants (BS, CS, PS, AS). Across 650 held‐out test plans, DS achieved the lowest overall voxel‐wise MAE/RMSE, while BS exhibited voxel‐wise errors essentially comparable to DS and CS, PS, and AS showed small increases in voxel‐wise errors. These findings indicate that adding auxiliary supervision did not consistently improve overall dose‐prediction accuracy.

At the endpoint level, however, different auxiliary tasks produced different effects. Under the composite‐loss setting, BS showed more consistent cross‐seed improvements in several endpoints, including PTV D95, PTV mean dose, spinal cord maximum dose, and LungL V20, whereas the effects of the other configurations were more mixed. This suggests that auxiliary supervision may affect specific dose characteristics without substantially changing global voxel‐wise error.

This difference also reflects the information captured by different evaluation metrics. MAE and RMSE summarize global dose‐distribution error, whereas DVH endpoints focus on dose behavior within specific anatomical structures. Therefore, similar global errors do not necessarily imply similar clinically relevant endpoint performance.

These observations are also relevant when considering the relative merits of DS and BS. DS showed a slight advantage in global voxel‐wise accuracy, whereas BS exhibited smaller mean biases in several dosimetric endpoints. However, the stronger optimization coupling observed for Beam supervision did not consistently translate into improved dose‐prediction performance, and the endpoint‐level benefits of BS varied with the primary dose‐loss formulation. Therefore, the present results do not establish a clear overall preference for either configuration; the choice between DS and BS should instead be considered in relation to the evaluation objectives and training conditions.

Residual deviations remained for endpoints such as PTV coverage and spinal cord maximum dose. One possible contributing factor is heterogeneity in the reference dose distributions. In particular, a subset of plans showed substantially lower PTV D95 values than the majority of cases, while spinal cord maximum dose also varied considerably across patients. Residual discrepancies may also reflect limitations of the composite dose objective, in which the differentiable DVH term was optimized jointly with voxel‐wise and spatial‐consistency components rather than directly targeting individual clinical endpoints. Because the relative contribution of these terms was governed by a predefined weighting scheme, improvement in the overall training objective does not necessarily ensure exact agreement for every endpoint. As no stratified analysis or dedicated loss‐component ablation was performed, the relative contributions of these factors cannot be determined from the present study.

### 4.2 Auxiliary Tasks Influence Dose Prediction Through Different Pathways

The auxiliary tasks differed in how they were connected to DoseNet. Beam and Center both received the predicted dose and therefore formed a direct computational pathway to DoseNet. Their degree of coupling, however, was different. Beam supervision consistently produced non‐zero gradients with larger relative magnitudes than Center supervision. In addition, CT + predicted dose outperformed CT‐only for Beam prediction across all three seeds, indicating that the predicted dose provided stable additional information. Center supervision showed a weaker direct gradient and no consistent benefit from the predicted‐dose input. This indicates that the existence of an input connection does not necessarily mean that the auxiliary task consistently uses that information. The stronger coupling and more consistent predicted‐dose dependence observed for Beam may partly reflect its closer physical relationship to dose formation. These findings should therefore not be interpreted as evidence that Beam supervision is intrinsically superior to other auxiliary‐task designs.

The Patient task represents a different pathway. Because it receives CT alone, its auxiliary loss does not directly propagate gradients to DoseNet. Although the Patient loss did not directly backpropagate to DoseNet, PS still showed changes in endpoint behavior relative to DS under the MSE‐only setting. This does not imply a direct Patient‐to‐DoseNet optimization effect, because enabling the Patient task altered the joint training configuration, including the set of active loss terms and the associated weighting and normalization schedule. In addition, anatomical structure masks were already supplied explicitly to M_Dose, so the Patient auxiliary task may have introduced partly redundant anatomical supervision. Its limited or inconsistent effect in the present framework therefore does not imply that anatomy‐related supervision is generally ineffective, particularly in settings where anatomical information is not explicitly provided to the primary network.

These results therefore separate two questions: whether an auxiliary loss directly participates in DoseNet optimization, and whether the auxiliary task actually uses the additional information introduced by its design. Gradient analysis and input ablation provide evidence for these two aspects, respectively.

### 4.3 Auxiliary‐task Effects Depend on the Primary Dose Objective

The final effect of auxiliary supervision also depended on the primary dose‐loss formulation.

Under the composite‐loss setting, BS showed relatively consistent cross‐seed improvements across several endpoints. Under MSE‐only training, this consistency decreased. PS showed mixed effects under both loss formulations, although its seed‐wise consistency improved modestly for PTV D95 and PTV mean dose under MSE‐only training. CS and AS also showed changes between the two settings.

These findings indicate that the effect of an auxiliary task cannot be interpreted independently of the primary training objective. The same auxiliary task may lead to different endpoint behavior under different dose‐loss formulations, so an effect observed in one setting should not be considered an intrinsic property of the task itself.

The Beam results further show that stronger direct optimization coupling does not necessarily translate into better performance. Under MSE‐only training, the normalized relative Beam‐gradient ratio remained substantial, whereas the consistency of BS endpoint improvements became weaker. Thus, relative gradient magnitude characterizes direct optimization coupling within the corresponding training objective rather than guaranteeing final performance benefit.

Overall, auxiliary‐task effects should be evaluated not only by how a task is connected to the primary network, but also by whether its performance effect remains stable across training objectives and random seeds.

### 4.4 Limitations and Implications

Several limitations define the scope of generalization of this work. First, the auxiliary networks were independently parameterized rather than implemented within a shared‐encoder multi‐task framework, so the conclusions mainly apply to the present auxiliary‐supervision design. Second, gradient analysis characterizes direct optimization coupling with DoseNet but does not fully explain final performance changes. Third, the CT + predicted dose versus CT‐only comparison evaluates whether predicted dose provides additional information, but does not further separate the contribution of individual input sources. Finally, only three random seeds were evaluated, and multiple test plans originated from the same patient anatomy; larger independent cohorts are therefore needed for further validation.

Taken together, these findings highlight three complementary aspects of auxiliary supervision: optimization coupling, information use, and performance robustness. Gradient analysis assesses whether an auxiliary objective directly participates in primary‐network optimization, input ablation tests whether the added information is actually used, and cross‐seed and cross‐loss comparisons assess whether performance effects are stable. These three forms of evidence are complementary and provide a more complete interpretation of auxiliary supervision than simply comparing model performance before and after adding an auxiliary task.

These findings may also provide practical considerations for the design and evaluation of auxiliary supervision in future dose‐prediction studies. In particular, gradient analysis can help examine whether an auxiliary loss establishes the intended direct optimization pathway to the primary network, while input ablation can assess whether additional predicted‐dose information benefits the auxiliary prediction task. However, neither stronger optimization coupling nor improved auxiliary‐task prediction necessarily indicates better dose‐prediction performance, which should still be evaluated using the relevant dose metrics and training conditions. Beyond these analyses, auxiliary‐task prediction outcomes may also have potential interpretive value. For example, poor Beam prediction might help identify inaccurate dose predictions if a reliable relationship between the two were established. The present results do not establish such a relationship, and therefore do not support using Beam prediction performance alone to determine the reliability of a dose prediction.

## 5 CONCLUSION

In this study, we investigated how heterogeneous auxiliary objectives relate to dose‐prediction performance for left‐sided breast IMRT under controlled experimental conditions. Using an independently parameterized auxiliary‐supervised joint optimization framework trained without encoder sharing, we compared five task configurations DS, BS, CS, PS, and AS on 650 test plans from held‐out patients.

The results showed that auxiliary supervision did not consistently reduce global voxel‐wise dose error. DS achieved the lowest MAE/RMSE, while BS remained comparable, and the different auxiliary tasks produced distinct effects at the dosimetric endpoint level. Further analyses revealed that Beam, Center, and Patient interacted with DoseNet through different pathways. Beam and Center both formed direct optimization connections through the predicted dose, but Beam showed stronger gradient coupling and more consistent use of predicted‐dose information, whereas Patient had no direct auxiliary‐gradient pathway to DoseNet.

The final performance effects of auxiliary supervision were also not fixed. When the primary dose objective was changed from the composite loss to MSE‐only, the cross‐seed endpoint patterns of the auxiliary configurations changed substantially, indicating that auxiliary‐task effects depend on the primary training objective and should not be inferred from a single loss setting alone.

Overall, these results suggest that auxiliary supervision should be evaluated along three distinct dimensions: optimization coupling, information use, and performance robustness. Final performance comparisons alone are insufficient to fully characterize the role of an auxiliary task. Combining gradient analysis, input ablation, and performance comparisons across random seeds and primary loss formulations provides a more complete framework for the design and evaluation of auxiliary supervision in radiotherapy dose prediction.

## AUTHOR CONTRIBUTION

**Yusi Xie**: Conceptualization; data curation; formal analysis; funding acquisition; methodology; software; validation; visualization; writing—original draft; writing—review & editing. **Haiming Yang**: Formal analysis; validation; writing—review & editing. **Chengxian Ma**: Funding acquisition; writing—review & editing. **Wenjie Liang**: Data curation; writing—review & editing. **Li Mo**: Data curation; writing—review & editing. **Liuke Liang**: Data curation; writing—review & editing. **Qingguo Fu**: Conceptualization; methodology; supervision; writing—original draft; writing—review & editing.

## CONFLICT OF INTEREST STATEMENT

The authors declare no conflicts of interest.

## ETHICS STATEMENT

This retrospective study was approved by the Guangxi Medical University Cancer Hospital ethics committee (Grant No. KY2023625). All data were de‐identified prior to analysis. Given the retrospective nature of the study and the exclusive use of anonymized imaging and dosimetric information, the requirement for informed consent was waived by the institutional review board.

## Supporting information




Supporting Information

## Data Availability

Authors will share data upon request to the corresponding author.
