# Supplementary material for: Impact of Auxiliary Task Heterogeneity on Dose Prediction: A Controlled Study of Optimization Coupling and Dose Outcomes
Source: J Appl Clin Med Phys. 2026 Nov 9;27(11):e70906. doi: 10.1002/acm2.70906 (PMC13748823; doi:10.1002/acm2.70906)
Supplement: Supplementary file 1 [file ACM2-27-e70906-s001.docx]

# Supplement A. Data, cohort, and ethics

## A.1 Patient Cohort and Treatment Characteristics

A total of 30 patients with left-sided breast cancer treated at a tertiary cancer center between May 2019 and January 2023 were retrospectively included in this study. Inclusion criteria were as follows: (1) a single-isocenter treatment plan; (2) complete and artifact-free planning CT images; (3) complete structure delineation, including target volumes, contralateral breast, heart, bilateral lungs, spinal cord, and body contour; and (4) a prescribed dose of 50 Gy delivered in 25 fractions; (5) treatment planned and delivered using intensity-modulated radiotherapy (IMRT).

Patients were excluded if they had multi-isocenter treatment plans, overlapping radiotherapy plans, incomplete CT images, or missing structure delineations. All patients were treated using intensity-modulated radiotherapy (IMRT) according to institutional clinical protocols.

Both breast-conserving surgery and mastectomy cases were included in the cohort. The study did not explicitly stratify patients according to surgical type or lymph node irradiation status. Instead, anatomical and treatment-related variations were implicitly encoded through the planning target volumes (PTVs), organ-at-risk delineations, and beam configurations provided as model inputs.

Although the ground-truth (GT) dose distributions were not directly extracted from delivered clinical treatment plans, they were generated under clinically consistent prescriptions and planning constraints. Importantly, all target volumes and organs at risk were delineated by physicians, ensuring that variations in target geometry and spatial relationships—such as those arising from different surgical procedures—were inherently preserved in the GT dose distributions.

## A.2 Image Acquisition, Contouring, and Quality Assurance

Planning CT images were acquired using a GE LightSpeed RT scanner (GE Healthcare). Due to routine clinical practice over multiple years, CT acquisition parameters were not fully uniform across patients. The in-plane resolution ranged from approximately 0.95 × 0.95 mm² to 1.27 × 1.27 mm², while the slice thickness was consistently 5 mm for all cases. All CT images were resampled to a uniform voxel spacing prior to model development, with preprocessing details described in a separate supplementary section.

All target volumes and organs at risk were delineated in the MIM software (MIM Software Inc.) by radiation oncologists with attending physician qualifications or higher. These clinician-approved structure delineations were used as anatomical inputs for the dose prediction framework.

Clinical treatment planning and dose calculation for patient care were originally performed by certified medical physicists using the Pinnacle 3.0 treatment planning system (Philips Healthcare), and all clinical plans underwent routine institutional quality assurance and review. However, the clinically delivered Pinnacle dose distributions were not used as ground truth in this study. Instead, ground-truth dose distributions were generated independently using an automated planning workflow implemented in the matRad treatment planning framework, as described in Supplementary Methods.

## A.3 Ethical Approval and Patient Privacy Protection

This retrospective study was approved by the institutional ethics committee (approval number withheld for review). As the study exclusively used anonymized imaging and dosimetric data and did not involve identifiable personal information, the requirement for informed consent was waived by the institutional review board.

## A.4 Data Availability Statement

The patient-level datasets generated and/or analyzed during the current study are not publicly available due to institutional data governance policies. Although the data were anonymized for research use, patient-level imaging and structure data cannot be shared publicly. Access to the anonymized data may be considered upon reasonable request to the corresponding author, subject to institutional authorization and applicable ethical approvals.

# Supplement B. Plan Generation with Geometry Perturbations

## B.1 Factorized Plan Perturbation Strategy

To systematically evaluate the robustness and task interactions of the proposed auxiliary-supervised dose prediction framework, we designed a factorized plan perturbation strategy that decomposes geometric variability into two independent components: beam direction perturbations and isocenter perturbations. These two factors were perturbed separately and then combined to generate a structured yet controlled set of treatment plan configurations for each patient.

This design allows us to explore model behavior under realistic but limited geometric variations while avoiding large replanning heterogeneity that could confound the analysis of task interference and representation learning.

For each of the 30 patients, 13 beam-angle configurations and 10 isocenter locations were combined, resulting in 130 plan parameter sets per patient (13 × 10).

### B.1.1 Beam Angle Perturbations

In this study, controlled perturbations of beam field angles were introduced as part of the data generation process to examine the behavior of the proposed auxiliary-supervised joint optimization learning framework under limited, clinically plausible variations in beam geometry. The purpose of these perturbations was not to induce large dose differences or to optimize plan quality, but rather to introduce mild geometric variability while preserving overall plan consistency. This design allows the evaluation of model stability and task interaction under constrained beam-geometry changes without introducing excessive heterogeneity that could confound the learning process.

Selection of the Base Field Angle Combination

A reference field angle combination was first defined based on the most frequently observed beam configurations in the original clinical treatment plans of the 30 patients. This reference configuration represents a commonly used clinical setup and serves as a stable baseline for subsequent perturbations. The six selected gantry angles (300°, 330°, 10°, 110°, 135°, and 160°) were chosen according to their prevalence in the original plans, ensuring that the baseline geometry reflects routine clinical practice rather than an artificially constructed configuration.

Controlled Angle Perturbations

Starting from the reference configuration, small angular perturbations were applied independently to individual fields, with a maximum deviation of ±10°. These perturbations were intentionally limited in magnitude to maintain comparable dose characteristics across plans while introducing localized variations in beam geometry. Such controlled variations are representative of minor planning-level adjustments rather than substantial replanning or alternative treatment strategies.

A total of 13 field angle configurations, including the reference setup and its perturbed variants, were generated for each patient. The complete set of field angle combinations is listed below:

• 300°, 330°, 10°, 110°, 135°, 160°

• 290°, 330°, 10°, 110°, 135°, 160°

• 300°, 320°, 10°, 110°, 135°, 160°

• 300°, 330°, 0°, 110°, 135°, 160°

• 300°, 330°, 10°, 100°, 135°, 160°

• 300°, 330°, 10°, 110°, 125°, 160°

• 300°, 330°, 10°, 110°, 135°, 150°

• 310°, 330°, 10°, 110°, 135°, 160°

• 300°, 340°, 10°, 110°, 135°, 160°

• 300°, 330°, 20°, 110°, 135°, 160°

• 300°, 330°, 10°, 120°, 135°, 160°

• 300°, 330°, 10°, 110°, 145°, 160°

• 300°, 330°, 10°, 110°, 135°, 170°

Because the angular perturbations were intentionally small, the resulting dose distributions across these configurations are expected to exhibit limited variability in conventional dose-volume metrics. Rather than being a limitation, this behavior reflects the intended design of the perturbation strategy, which aims to preserve clinical comparability while enabling the assessment of model robustness to mild beam-geometry changes. This controlled setting allows investigation of whether auxiliary beam-related tasks can be integrated into the learning framework without destabilizing dose prediction under small geometric variations.

Larger perturbations (e.g., ±20° or ±30°), which may lead to more pronounced dose differences and clinically distinct planning scenarios, were not explored in the present study. Such conditions introduce additional sources of variability that fall outside the scope of the current robustness-oriented design and will be investigated in future work.

### B.1.2 Isocenter Perturbations (Spatial Anchor Variability)

While beam angle perturbations probe sensitivity to directional changes, isocenter perturbations were introduced to address a different and complementary failure mode: the risk that the model may rely on trivial geometric shortcuts, such as a fixed spatial correspondence between the isocenter and the planning target volume (PTV).

To mitigate this risk and encourage the model to learn anatomically grounded dose mappings rather than absolute spatial heuristics, we intentionally decoupled the isocenter location from a single patient-specific configuration.

Isocenter Sampling Strategy

For each patient, 10 isocenter locations were randomly sampled from the set of clinically observed isocenter positions across the entire cohort of 30 patients. This approach avoids arbitrary or unrealistic spatial shifts while ensuring that all sampled isocenters correspond to plausible clinical setups.

Each patient’s original isocenter served as the baseline, and the sampled isocenter positions introduced spatial variability that disrupts a fixed correspondence between isocenter coordinates and patient anatomy.

An example set of 10 sampled isocenter locations (in mm) is shown below:

• (342.2, 288.1, 215)

• (345.0, 246.0, 175)

• (366.0, 266.0, 170)

• (391.0, 263.0, 235)

• (396.1, 307.2, 215)

• (366.2, 279.3, 180)

• (416.4, 289.4, 240)

• (347.4, 255.0, 235)

• (370.1, 258.5, 230)

• (355.0, 216.6, 235)

These perturbations are not intended to represent realistic setup errors within a single patient, but rather to introduce controlled spatial diversity that prevents the model from collapsing onto isocenter-centered shortcuts.

### B.1.3 Combined Plan Generation and Design Rationale

By combining 13 beam-angle configurations with 10 isocenter locations, we generated 130 plan parameter sets per patient, yielding a total of 3,900 plans across the cohort. After data integrity screening, a small number of plans were excluded due to file corruption, yielding a final dataset of 3,863 plans used for model development and evaluation (details provided in Section B.2). All generated plans were optimized using identical dose calculation settings, optimization algorithms, and objective definitions (see Section B.1.4). This ensures that observed differences in dose distributions arise solely from controlled geometric perturbations rather than from optimization variability.

This factorized design enables the joint evaluation of two complementary robustness dimensions:

• Directional robustness, assessed through small beam angle perturbations that preserve the underlying physics of dose deposition.

• Spatial robustness, assessed through isocenter perturbations that break fixed geometric correlations and encourage anatomically meaningful representations.

Crucially, these two perturbation axes target distinct failure modes. Their combination allows the model to be evaluated in a low-dimensional but structured geometric variation space, providing a rigorous assessment of task interactions and generalization behavior without introducing excessive plan heterogeneity.

While larger perturbation ranges may induce more pronounced dose differences, the present study intentionally focuses on clinically comparable plan variants to isolate the effects of auxiliary task supervision. Future work will explore larger perturbation ranges and more systematic sampling strategies to further investigate dose variability and clinical impact.

### B.1.4 Plan Optimization and Dose Generation (matRad)

All treatment plans were generated using the open-source matRad treatment planning system (version 2.10.0). To ensure a controlled comparison across different task configurations, identical dose calculation settings, optimization algorithms, and objective definitions were used for all plans. The only sources of variation between plans were factorized plan perturbations, consisting of different beam angle combinations and perturbed isocenter locations for each patient.

All plans were created using 6 MV photon beams with a source-to-axis distance (SAD) of 1000 mm and a source-to-collimator distance (SCD) of 500 mm. Dose calculation was performed using the pencil beam algorithm implemented in matRad. The dose calculation grid resolution was fixed at 4 × 4 × 4 mm³, and the bixel width was set to 5 mm for all plans.

Each plan was prescribed a total dose of 50 Gy delivered in 25 fractions. Dose–volume objectives and organ-at-risk (OAR) constraints were defined according to the Chinese Medical Doctor Association (CMDA) guidelines for left-sided breast cancer radiotherapy (2020 Edition). Table S1 and S2 summarize clinical planning goals and corresponding dose–volume objectives used for matRad-based plan generation respectively.

**Table S1. Clinical dose constraints (CMDA 2020)**

| **Organ** | **Dose constraint** |
| --- | --- |
| Heart | Dmean < 8 Gy (10 Gy); V5 < 40% (50%) |
| Left Lung | Dmean < 15 Gy (16 Gy); V20 < 30% (35%); V5 < 50% (55%) |
| Right Lung | V5 < 20% (25%) |
| Right Breast | Dmean < 5 Gy (8 Gy) |
| Spinal Cord | Dmax < 40 Gy (45 Gy) |

**Table S1** summarizes the clinical dose constraints applied to major organs at risk (OARs), defined according to the Chinese Medical Doctor Association (CMDA) guidelines for left-sided breast cancer radiotherapy (2020 Edition). Values in parentheses indicate upper tolerance limits acceptable under exceptional clinical circumstances.

**Table S2. Dose–volume objectives implemented in matRad (cst structure)**

| **VOI name** | **VOI Type** | **OP** | **Function** | **p** | **Parameters** |
| --- | --- | --- | --- | --- | --- |
| ptv | TARGET | 1 | Squared Deviation | 10 | d_ref_ = 50 |
| heart | OAR | 2 | Mean Dose | 1 | d_ref_ = 8 |
| heart | OAR | 2 | Max DVH | 1 | d=5,v_max_=40 |
| lung_l | OAR | 2 | Mean Dose | 1 | d_ref_=15 |
| lung_l | OAR | 2 | Max DVH | 1 | d=20,v_max_=30 |
| lung_l | OAR | 2 | Max DVH | 1 | d=5,v_max_=50 |
| lung_r | OAR | 2 | Max DVH | 1 | d=5,v_max_=20 |
| breast_r | OAR | 2 | Mean Dose | 1 | d_ref_=5 |
| cord | OAR | 2 | Min/Max dose constraint | 1 | d_min_=0,d_max_=40 |

**Notes**

a) VOI denotes volume of interest; TARGET and OAR correspond to planning target volumes and organs at risk, respectively.
b) OP indicates overlap priority used to resolve voxels shared by multiple VOIs (lower OP values indicate higher priority).
c) *Function* specifies the objective or constraint class implemented in matRad (e.g., Squared Deviation, Mean Dose, DVH-based constraint, or Min/Max dose constraint).
d) *p* denotes the penalty weight applied to each objective in the weighted-sum optimization formulation.
e) For Squared Deviation objectives, d_ref_ denotes the prescribed reference dose (Gy).
f) For DVH-type constraints, *d* denotes the dose threshold (Gy) and v_max_ denotes the upper bound on the fractional or percentage volume.
g) All dose thresholds are specified in Gy, and DVH volumes are specified in %.
h) All treatment plans were prescribed a total dose of 50 Gy delivered in 25 fractions, consistent with the clinical protocol used to define the planning goals.

**Table S2.** lists the corresponding dose–volume objectives and constraints implemented in matRad via the cst structure for inverse plan optimization.

Plan optimization was formulated as a constrained nonlinear optimization problem, where multiple dose objectives were combined into a single weighted objective function subject to structure-specific constraints. Optimization was performed using the default IPOPT solver integrated in matRad via the matRad_OptimizerIPOPT interface, which is designed for large-scale nonlinear optimization.

This study focuses exclusively on the planning and dose optimization stage. Mechanical delivery parameters (e.g., collimator motion and machine-specific delivery constraints) were not modeled, as the objective of this work was to investigate dose prediction under controlled planning geometries rather than treatment delivery.

Although the ground-truth (GT) dose distributions used in this study were not directly extracted from delivered clinical treatment plans, they were generated using a clinically established inverse planning framework (matRad) under guideline-consistent prescriptions and dose–volume constraints. All planning target volumes (PTVs) and organs at risk (OARs) were delineated by physicians and directly inherited from real patient data. Consequently, patient-specific anatomical geometry, spatial relationships between targets and OARs, and clinically relevant structural variability were preserved in the GT dose generation process.

The use of matRad-based planning enabled systematic and programmatic control over beam geometry, isocenter configuration, and optimization objectives within a unified and transparent optimization framework. Compared with retrospective clinical plans produced by different planners, institutions, or treatment planning systems, this approach allows geometric and task-related factors to be isolated while minimizing confounding variability introduced by planner-dependent decisions.

Importantly, the role of matRad in this study is not to enable exact reproduction of patient-specific treatment plans, but to provide a planning-consistent and methodologically transparent reference framework for generating GT dose distributions under controlled conditions. While patient-level imaging and structure data cannot be publicly released due to institutional restrictions, the complete planning pipeline—including optimization settings, objective definitions, and perturbation strategies—is fully specified and implemented in open-source code. This design supports procedural reproducibility and transferability of the experimental methodology to independent datasets, rather than data-level replication of individual cases.

Accordingly, the synthetic GT dose distributions should be interpreted as controlled planning-based reference doses rather than surrogates for delivered treatment doses. This approach is well suited for investigating model robustness, task interference, and geometric generalization in learning-based dose prediction, and is consistent with prior studies that employ planning-based dose generation to study algorithmic behavior under controlled experimental settings.

## B.2 Data Integrity and Exclusion Criteria

During data preprocessing, all generated treatment plans underwent automated integrity checks. A total of 37 plans were excluded due to corrupted or incomplete data files that could not be reliably parsed or processed. After exclusion, 3,863 plans remained and were used for subsequent analysis, including 2,831 plans for training, 382 for validation, and 650 for testing.

Although a small fraction of generated plans was excluded due to technical file corruption, these exclusions were non-selective and unrelated to patient anatomy, treatment intent, or optimization settings. As a result, the patient-level composition and the predefined training/validation/test splits were preserved. The balance of anatomical characteristics and key dosimetric metrics across dataset splits was assessed at the patient level and is reported in Supplement D. No exclusion criteria were applied based on dosimetric values or anatomical characteristics.

# Supplement C. Preprocessing and label construction

## C.1 Resampling and interpolation

To ensure spatial consistency across patients and modalities, all CT images, dose distributions, and organ-at-risk (OAR) masks were resampled to a unified voxel grid prior to model training and evaluation. A consistent resampling strategy was applied to all data types to preserve the spatial correspondence between anatomical structures and dose information.

Let the original voxel spacings along the x, y, and z directions be denoted as $x,y,z$, and the corresponding numbers of voxels be $\text{cubeDim}_{x}$, $\text{cubeDim}_{y}$, and $\text{cubeDim}_{z}$. Given the target voxel spacings $\text{targetSize}_{x}$, $\text{targetSize}_{y}$, and $\text{targetSize}_{z}$, the transformation coefficients for each direction were computed as:

$\text{tranf}_{d}=\frac{\text{cubeDim}_{d}\cdot d}{\text{targetSize}_{d}},d\in\{x,y,z\}$ (5)

Based on these coefficients, the resampling coordinate sequences for each axis were defined as:

$X_{\text{in}},Y_{\text{in}},Z_{\text{in}}=1:\frac{\text{tranf}_{d}}{d}:\text{cubeDim}_{d},d\in\left\{ x,y,z \right\}$ (6)

Three-dimensional linear interpolation was then applied to resample the CT images, dose data, and OAR masks using the same spatial transformation framework. This unified interpolation ensured consistent spatial alignment across modalities and avoided discrepancies introduced by modality-specific resampling procedures. In this study, all resampled volumes were standardized to a size of $128\times128\times128$.

The original CT image dimensions varied across patients, with in-plane sizes ranging from 511 to 512 voxels along both the x and y directions, and the number of axial slices ranging from 73 to 124 along the z direction. Resampling to a fixed target size enabled uniform input dimensionality for subsequent model processing while preserving relative anatomical geometry.

For OAR masks, interpolation was applied within the same coordinate system to maintain voxel-wise correspondence with CT images and dose distributions. The resampled masks were used consistently throughout training and evaluation to ensure coherent spatial supervision.

All resampling operations were implemented using the interpn function in MATLAB with linear interpolation. This choice provided a balance between numerical stability and computational efficiency while maintaining consistency across all data modalities. Further details on the interpolation framework are available in the matRad documentation.

## C.2 CT Normalization

To standardize CT inputs across patients and facilitate stable model training, all CT volumes were preprocessed using a unified intensity normalization procedure prior to network input.

First, CT voxel values were clipped to a fixed Hounsfield Unit (HU) range of [-1000.1000], which encompasses the vast majority of clinically relevant tissue densities in the thoracic region, including lung, adipose tissue, and soft tissue. Values outside this range are typically associated with air cavities, dense bone, metallic artifacts, or noise and were therefore truncated to limit the influence of extreme outliers.

Following clipping, CT values were linearly rescaled to the range [0,1] using min–max normalization:

$\mathrm{CT}_{\mathrm{norm}}=\frac{\mathrm{CT}_{\mathrm{clip}}+1000}{2000}$ (7)

This normalization step ensures consistent input scaling across patients and improves numerical stability during network optimization.

The same preprocessing strategy was applied consistently to all cases. As this study focuses on learning spatial dose–geometry relationships rather than CT intensity modeling, the adopted clipping and normalization procedure serves as a standardized and widely used preprocessing step rather than a task-specific optimization.

## C.3 Geometric Input Encoding and Smoothing (Structures, Isocenter, and Beams)

In addition to CT, our model uses several geometry-related input channels to represent structures, isocenter configuration, and beam direction in a unified tensor format. These channels are designed as computational encodings that provide spatial priors for learning-based dose prediction. Importantly, they are not intended to replicate the full physical delivery process (e.g., detailed MLC modulation), but rather to provide compact and controllable geometric descriptors that are consistent across all generated plans.

Because these geometric channels originate from discrete voxelized representations (binary masks or rasterized fields), direct use of hard 0/1 boundaries can introduce aliasing and high-frequency artifacts that are not informative for learning. Therefore, we applied mild smoothing/softening operations to obtain numerically stable “soft” representations:

Structure masks were blurred with a small Gaussian kernel ($\sigma=1$) to reduce staircase artifacts and provide continuous boundary cues while preserving gross anatomy.

Isocenter information was represented by a distance-based radial encoding derived from the Euclidean distance map (computed in MATLAB within the Body region) and transformed into a smooth field using a Gaussian radial basis function with $\sigma=10$.

Beam geometry was encoded as slice-wise rasterized beam masks and smoothed with a moderate Gaussian blur ($\sigma=2$) to obtain soft beam envelopes.

These transformations are applied consistently across training, validation, and testing, ensuring that the model always observes the same type of input representation. We note that such encodings may introduce inductive biases (and, in our case, the isocenter-related encoding was observed to correlate negatively with performance in certain settings), which is explicitly discussed in the main text as part of the task-selection analysis. The purpose of documenting these steps here is to ensure transparency and reproducibility of the input construction pipeline.

### C.3.1 Structure Mask Smoothing

To reduce voxel-level discretization artifacts introduced by resampling and to obtain a consistent structure representation throughout the pipeline, a mild smoothing operation was applied to the structure masks.

Specifically, each binary mask was convolved with a three-dimensional Gaussian kernel (σ = 1 voxel), producing a softly varying boundary representation while preserving the overall structure shape and spatial extent. This smoothing was applied uniformly to all cases and to all structures, and the same smoothed masks were used consistently wherever structure masks were required (including model inputs and subsequent structure-restricted metric calculations). Using a unified mask representation avoids potential train–test or preprocessing–evaluation mismatches and improves numerical stability for boundary-sensitive computations.

The smoothing strength (σ = 1) was intentionally kept small and should be interpreted as a boundary regularization step rather than a modification of anatomical definitions. Because the operation only affects a narrow band around the original binary boundary, its impact is primarily on voxel assignment near edges, while the interior of each structure remains unchanged. This choice was adopted to improve robustness against jagged boundaries caused by grid discretization, rather than to optimize any specific metric.

### C.3.2 Generation of Isocenter Distance Map

During plan generation, a voxel-wise isocenter distance map (centerMatrix) was constructed in MATLAB to explicitly encode the spatial relationship between each voxel and the treatment isocenter. For each patient and plan configuration, the Euclidean distance from the isocenter $\left( x_{c} , y_{c} , z_{c} \right)$to every voxel $\left( i , j , k \right)$was computed as

$D(i,j,k)=\sqrt{\left( x_{c}-i)^{2}+(y_{c}-j)^{2}+(z_{c}-k)^{2} \right.}$ (8)

To restrict the distance definition to anatomically relevant regions, the resulting distance field was multiplied by the body mask, such that distances outside the patient body were excluded. This procedure yielded a body-constrained isocenter distance matrix on the same resampled $128\times128\times128$ grid as the CT and dose data.

During model preprocessing in Python, this distance matrix was further transformed into a bounded proximity representation using a Gaussian-shaped encoding:

$\text{Center}_{\text{enc}}\left( v \right)=\exp\left( -\frac{D(v)^{2}}{2\sigma^{2}} \right)$ (9)

where $D(v)$denotes the Euclidean distance value stored in centerMatrix at voxel $v$, and $\sigma$controls the spatial decay of the encoding. In this study, $\sigma$was set to 10 voxels for all experiments. This transformation maps voxels closer to the isocenter to values near 1 and smoothly attenuates the contribution of voxels farther away, yielding a numerically stable and continuous geometric input channel.

The Gaussian encoding does not alter the relative ordering of distances but serves to compress the dynamic range of the distance values and facilitate learning in the neural network. The same isocenter distance definition and encoding parameters were applied consistently across all patients, beam-angle configurations, and task settings.

### C.3.3 Beam Mask Generation

To encode beam-direction information as a structured input to the dose prediction network, we constructed a beam geometry mask volume for each incident beam angle. This volume is not intended to represent deliverable fluence modulation (e.g., MLC leaf motion or aperture shapes). Instead, it provides a coarse, programmatic spatial prior that indicates the approximate beam entry direction and its potential intersection region with the patient volume, serving as an auxiliary geometric descriptor.

For a given beam angle $\theta$, a central ray passing through the isocenter was defined. The ray was then laterally translated by $\pm20$cm to form two bounding rays, and the region between them was rasterized into a binary mask for each axial slice $z$. Repeating this procedure across all slices yielded a 3D binary beam mask $B_{\theta}(x,y,z)$. The set of beam masks from the six beam angles was stacked to form a multi-channel beam-geometry representation.

The translation range ($\pm20$ cm) was selected to ensure that the constructed mask reliably covers the clinically relevant field-of-view around the isocenter for whole-breast irradiation across patients, while remaining a generic geometric envelope rather than a machine-specific delivery constraint. Therefore, this representation should be interpreted as a geometry encoding rather than an accurate simulation of linac mechanical limits.

Finally, to avoid aliasing artifacts from discretization and to provide smoother gradients for learning, each binary beam mask was converted into a soft beam mask using a Gaussian blur operation (kernel size = 5, $\sigma=2$), consistent with the preprocessing applied to other auxiliary geometric channels. This smoothing step improves numerical stability and makes the beam-channel representation less sensitive to voxel-level boundary discontinuities, while preserving the overall beam-directional pattern.

# Supplement D. Model architecture and training objective

## D.1 Network Architecture

This section describes the architectural design of the proposed framework, with emphasis on the principles guiding task decoupling, network instantiation, and task-specific interfaces. Figure S1 provides a schematic overview of the proposed network architecture, including branch-wise input/output definitions and channel compositions. The present section summarizes the architectural design principles and implementation details that are important for reproducibility but may not be fully explicit from the diagram. The goal of the architecture is not to maximize parameter sharing, but to enable a controlled analysis of heterogeneous auxiliary tasks and their interactions with dose prediction.

**Figure S1. Network architectures for dose prediction and auxiliary tasks.** (a) Main dose prediction network (M_Dose), implemented as a five-level 3D encoder–decoder with U-Net–style skip connections and progressive ×2 spatial downsampling. The network incorporates convolutional block attention modules (CBAM) at multiple encoder and decoder stages and an atrous spatial pyramid pooling (ASPP) block at the bottleneck to support multi-scale context aggregation. A Softplus activation is applied at the output to enforce non-negativity of the predicted dose volume. (b) Auxiliary task networks for patient structure prediction (M_Patient), beam-geometry prediction (M_Beam), and isocenter-related spatial prediction (M_Center). Each auxiliary network follows a 3D U-Net–based template with independent parameters and task-specific input/output configurations, as summarized in the subtask definition table. M_Patient predicts multi-organ structure masks from CT input alone, whereas M_Beam and M_Center take CT together with the predicted dose as inputs to produce geometry-related targets. No feature sharing is used between the main and auxiliary networks, and auxiliary supervision influences the main task only through loss-level coupling.

### D.1.1 Design principles and task decoupling

The proposed framework is designed as an analysis-oriented auxiliary-supervised joint optimization system to investigate how heterogeneous auxiliary tasks influence dose prediction under controlled conditions. Unlike conventional MTL architectures that rely on shared encoders, this work deliberately avoids feature sharing across tasks.

This design choice is motivated by the heterogeneous nature of the auxiliary tasks considered in left-sided breast IMRT, including anatomical structures, beam geometry, and isocenter positioning. These tasks encode distinct physical and geometric factors of dose formation and may induce gradient competition or negative transfer when forced into a shared representation space. To mitigate such interference and isolate task effects, all tasks are implemented as fully decoupled networks with independent parameters. Task interaction is introduced exclusively at the loss level through joint optimization.

Accordingly, the framework focuses on evaluating task alignment and interference rather than pursuing maximal prediction accuracy via representation sharing.

### D.1.2 Overall architecture and backbone instantiation

The framework consists of four independent 3D U-Net–style networks: one main dose prediction branch (M_Dose) and three auxiliary branches targeting patient anatomy (M_Patient), beam geometry (M_Beam), and isocenter representation (M_Center). All branches adopt the same high-level encoder–decoder topology to ensure architectural parity and comparability across tasks. For clarity, the branch-wise input tensors and output targets referenced in this section follow the channel definitions shown in Fig. 1 of the main text.

Each network follows a 5-level encoder–decoder structure with channel progression $\left[ 8 , 16 , 32 , 64 , 128 \right]$at successive encoder stages. Downsampling is performed using stride-2 3D convolutions, and decoder stages upsample feature maps by a factor of two with skip connections linking corresponding encoder layers. Despite identical architectural templates, no parameters are shared between branches.

Differences between branches are confined to task-specific input channels and output heads, which are designed according to the supervision objectives of each task.

### D.1.3 Task interfaces and conditioning strategy

Each subnetwork is assigned task-specific inputs and outputs that reflect its intended supervision role within the framework.

The dose branch (M_Dose) receives a multi-channel input composed of the CT volume and planning-time priors available during both training and inference, including structure masks, beam masks, and isocenter-related representations. It outputs a single-channel continuous 3D dose distribution of size $128\times128\times128$.

The patient branch (M_Patient) takes CT as its sole input and predicts multi-channel anatomical structure representations, corresponding to smoothed organ-at-risk and target masks.

The beam branch (M_Beam) and center branch (M_Center) are conditioned on the predicted dose rather than ground-truth dose. Specifically, both branches receive a two-channel input formed by concatenating the CT and the predicted dose from M_Dose. The beam branch outputs multi-channel beam mask volumes, while the center branch predicts a continuous isocenter-related spatial representation.

This asymmetric conditioning strategy is intentional. By conditioning geometry-related auxiliary tasks on the predicted dose, the framework avoids trivial supervision pathways and enforces consistency between the learned dose distribution and auxiliary geometric priors. This design ensures that auxiliary losses act as soft constraints on the dose solution rather than as direct predictors.

### D.1.4 Dose-branch specialization and auxiliary capacity control

To accommodate the higher representational demands of dose prediction, additional architectural components are introduced exclusively in the main dose branch. Specifically, convolutional block attention modules (CBAM) are incorporated after selected encoder downsampling stages and decoder fusion blocks, and an atrous spatial pyramid pooling (ASPP) module is applied at the bottleneck to aggregate multi-scale contextual information. These components are commonly used in volumetric prediction tasks to enhance spatial context modeling and are applied only to the dose branch in this study.

In contrast, auxiliary branches are implemented with a streamlined decoder design and do not include attention or multi-scale aggregation modules. This asymmetry reflects an explicit design constraint rather than an optimization objective. The auxiliary networks are intended to act as task-specific supervisors that provide geometric or anatomical consistency signals, rather than as competing predictors with comparable representational capacity to the dose branch.

Importantly, this architectural choice is made to limit variability introduced by auxiliary model capacity when comparing different task configurations. By keeping auxiliary branches lightweight and structurally consistent across experiments, differences observed between task settings can be more directly attributed to the presence or absence of auxiliary supervision, rather than to differences in network expressiveness. The study does not claim that this allocation is optimal, nor that increased auxiliary capacity would necessarily degrade or improve performance; rather, the design serves to maintain a controlled and interpretable experimental setting for analyzing task interactions.

### D.1.5 Output conventions and joint optimization overview

All subnetworks apply a Softplus activation at the final layer. For the dose branch, this enforces non-negativity of predicted dose values. For auxiliary branches, Softplus provides a smooth nonlinearity compatible with non-negative supervision targets such as smoothed structure masks, beam masks, and distance-field–style representations.

The four networks are trained jointly under a weighted sum of task losses without any parameter sharing. Auxiliary tasks influence the optimization process exclusively through loss-level coupling. In addition, a PTV-based regularization term is applied to the dose branch to penalize excessive intra-target dose variance with a warm-up schedule. Detailed loss formulations, weighting strategies, and optimization schedules are described in Section D.2. All architectural components were implemented in PyTorch and are fully specified in the released source code.

## D.2 Loss functions and task weighting

### D.2.1 Task configurations and loss-level task selection

Five task configurations were evaluated in this study: DS (dose only), BS (dose + beam), CS (dose + center), PS (dose + patient), and AS (all tasks). All configurations share the same network architecture, loss definitions, and optimization strategy. Task selection is implemented purely at the loss level.

Specifically, each task corresponds to a predefined loss term. When a task is disabled, its corresponding loss weight is set to zero, and the remaining task weights are re-normalized to maintain a comparable overall loss scale. No loss terms are redefined, removed, or modified across configurations. This design ensures that differences between task configurations arise from the presence or absence of task supervision, rather than changes in loss formulation or training intensity.

### D.2.2 Overall auxiliary-supervised joint optimization objective formulation

Let $\hat{D}\in\mathbb{R}^{128\times128\times128}$denote the predicted 3D dose volume and $D$its corresponding ground truth. Let $\hat{Y}^{pat}$, $\hat{Y}^{beam}$, and $\hat{Y}^{ctr}$denote the outputs of the patient-structure, beam-mask, and isocenter-distance auxiliary branches, with targets $Y^{pat}$, $Y^{beam}$, and $Y^{ctr}$, respectively.

The overall training objective is formulated as a weighted auxiliary-supervised loss:

$\mathcal{L}_{total}=\mathcal{L}_{mtl}+\lambda_{ptv}\cdot\rho_{ptv}\left( s \right)\cdot\mathcal{L}_{ptv\text{-}var}$ (10)

where $\mathcal{L}_{mtl}$aggregates the main dose-prediction loss and auxiliary task losses, and $\mathcal{L}_{ptv\text{-}var}$is a lightweight regularization term applied within the planning target volume (PTV). The warm-up factor $\rho_{ptv}(s)$gradually activates this regularizer during early training.

The same loss components, weighting strategy, and warm-up mechanism are used across all task configurations (DS, BS, CS, PS, AS). Different configurations are realized solely by enabling or disabling auxiliary losses via zeroing their corresponding weights, while the remaining weights are re-normalized to maintain a comparable overall loss scale. This design ensures that observed performance differences primarily reflect the effect of task supervision rather than changes in optimization strategy or training intensity.

### D.2.3 Auxiliary-supervised aggregation and dynamic weighting

The auxiliary-supervised loss $\mathcal{L}_{mtl}$ is defined as

$\mathcal{L}_{mtl}=w_{d}\mathcal{L}_{dose}+w_{p}\mathcal{L}_{patient}+w_{b}\mathcal{L}_{beam}+w_{c}\mathcal{L}_{center}$ (11)

where $\mathcal{L}_{dose}$is the primary objective, and the remaining terms correspond to auxiliary supervision.

To balance losses with different numerical scales, task weights are computed dynamically at each training step. A loss-proportional factor is first computed as

$\tilde{w}_{t}=\frac{\mathcal{L}_{t}}{\sum_{j} \mathcal{L}_{j}+\epsilon},t\in\{dose,patient,beam,center\}$ (12)

where $\epsilon={10}^{-6}$is a small constant introduced to ensure numerical stability. This factor is combined with a fixed task-importance prior $\hat{w}$ and a warm-up schedule applied only to auxiliary tasks:

$r_{t}(s)=\left\{ \begin{matrix} 1, & t=dose \\ min(1,s/(S\cdot\gamma)), & t\in\{patient,beam,center\} \end{matrix} \right.$ (13)

where $s$ denotes the training step, $S=1000$ is the ramp horizon, and $\gamma=0.2$. These values were fixed across all experiments and were not adjusted per task configuration.

The final normalized weights are

$w_{t}=\frac{\hat{w}_{t}\text{ }r_{t}(s)\text{ }\tilde{w}_{t}}{\sum_{j} \hat{w}_{j}\text{ }r_{j}(s)\text{ }\tilde{w}_{j}+\epsilon}$ (14)

This strategy gradually introduces auxiliary supervision while keeping the dose branch fully active from the start. The same weighting mechanism and warm-up schedule are applied uniformly across all task configurations, avoiding configuration-specific tuning.

### D.2.4 Main dose-prediction loss

Let $\hat{D},D\in\mathbb{R}^{128\times128\times128}$denote the predicted and reference 3D dose volumes, respectively. The prediction is constrained to be non-negative via a Softplus output, hence $\hat{D}_{i}\geq0$. The main dose loss is defined as a weighted sum of five complementary terms:

$L_{\text{dose}}=w^{\text{mse}}L_{\text{mse}}+w^{\text{edge}}L_{\text{edge}}+w^{z}L_{z}+w^{\text{wmse}}L_{\text{wmse}}+w^{\text{dvh}}L_{\text{dvh}}$ (15)

Each term targets a different numerical aspect of dose agreement (voxel-wise fidelity, spatial gradient consistency, inter-slice continuity, and ROI-level DVH behavior). The weights $w^{(.)}$are combined with the dynamic weighting strategy described in Section D.2.2.

**(a) Voxel-wise mean squared error**

$L_{\text{mse}}=\frac{1}{N}\sum_{i=1}^{N} (\hat{D}_{i}-D_{i})^{2}$ (16)

where $i$indexes voxels and $N$is the total number of voxels (i.e., $N={128}^{3}$).

**(b) 3D edge/gradient consistency loss**

We define a 3D gradient operator $\nabla(\cdot)$using fixed Sobel filters along the three spatial axes($x,y,z$). The edge loss is computed as an $\mathcal{l}_{1}$distance between the predicted and reference gradients:

$L_{\text{edge}}=\frac{1}{N}{\parallel\nabla\hat{D}-\nabla D\parallel}_{1}=\frac{1}{N}\sum_{i=1}^{N} \sum_{a\in\{x,y,z\}} \mid(\nabla_{a}\hat{D})_{i}-(\nabla_{a}D)_{i}\mid$ (17)

This term encourages consistency of spatial dose transitions (e.g., high-gradient regions) without directly optimizing any discrete clinical endpoint.

**(c) Axial inter-slice consistency loss**

Let $k\in\{1,\ldots,K\}$index axial slices (with $K=128$). Denote by $\hat{D}^{\left( k \right)}\in\mathbb{R}^{128\times128}$the predicted dose on slice $k$, and similarly $D^{\left( k \right)}$. The inter-slice loss matches slice-to-slice differences:

$L_{z}=\frac{1}{(K-1)\cdot128\cdot128}\sum_{k=1}^{K-1} {\parallel(\hat{D}^{\left( k + 1 \right)}-\hat{D}^{\left( k \right)})-(D^{\left( k + 1 \right)}-D^{\left( k \right)})\parallel}_{2}^{2}$ (18)

This term is used to discourage slice-wise discontinuities that may arise when learning 3D dose patterns.

**(d) Dose-weighted MSE**

To place greater emphasis on prediction accuracy in higher-dose voxels, we adopt a dose-weighted mean squared error. Let $\hat{D}$and $D$denote the predicted and ground-truth dose volumes, respectively. We define a voxel-wise weight map based on the normalized ground-truth dose:

$L_{\mathrm{wmse}}=\frac{1}{\mid\Omega\mid}\sum_{i\in\Omega} w_{i}(\hat{D}_{i}-D_{i})^{2}$ (19)

$w_{i}=1+\left( \frac{D_{i}}{D_{\max}} \right)\left( w_{\max} - 1 \right),D_{\max}=\underset{j\in\Omega}{max}D_{j}$ (20)

where $\Omega$denotes the voxel set (over the full 3D grid), $D_{\max}$is the maximum ground-truth dose within the current sample/batch tensor, and $w_{\max}$ controls the maximum up-weighting strength. This construction yields $w_{i}\in[1,\text{ }w_{\max}]$, so voxels near the highest-dose region receive the largest penalty. We set $w_{\max}=5$, which provides a moderate emphasis on higher-dose voxels while keeping the weighting bounded and numerically stable. Given the already large hyperparameter space in this study, $w_{\max}$was treated as a fixed implementation parameter rather than being exhaustively tuned.

**(e) Differentiable DVH matching loss.**

To encourage consistency in dose–volume behavior within each ROI, we compute a differentiable approximation of the DVH curve using a sigmoid-based soft threshold. For an ROI with voxel set $\Omega_{r}$ and dose threshold $d$, the predicted volume fraction receiving at least $d$Gy is approximated as

${\hat{\text{DVH}}}_{r}\left( d \right)\text{ }=\text{ }\frac{1}{\mid\Omega_{r}\mid}\sum_{\mathbf{v}\in\Omega_{r}} \sigma\text{ }\left( k\left( \hat{D}\left( \mathbf{v} \right)-d \right) \right)$ (21)

where $\sigma(\cdot)$is the logistic function and $k$controls the sharpness of the approximation. The DVH loss is defined as the L1 distance between predicted and reference DVH curves, summed over ROIs and dose bins:

$L_{\text{dvh}}\text{ }=\text{ }\sum_{r\in R} \frac{1}{\mid B\mid}\sum_{d\in B} \mid{\hat{\text{DVH}}}_{r}(d)-\text{DVH}_{r}(d)\mid$ (22)

where $r\in R$ indexes the set of regions of interest (ROIs) included in the DVH supervision, and $R$ consists of the planning target volume (PTV) and selected organs at risk (OARs) used throughout this study. In our implementation, dose thresholds are discretized as $B=\{d_{i}{\}}_{i=1}^{35}$uniformly spanning 0–70 Gy (i.e., torch.linspace(0,70,steps=35)), and we set $k=20$. These values were selected to provide a reasonably sharp yet numerically stable surrogate while keeping the computational cost tractable for 3D volumes; finer binning substantially increases memory/time due to repeated threshold evaluations.
For clarity, the same dose-loss components used for training are also aggregated into a fixed-weight validation score (val_metric_mae2) during hyperparameter selection. This metric does not alter the training objective and is not used for gradient updates.

### D.2.5 Auxiliary task losses

Auxiliary tasks are supervised using task-specific losses designed to provide geometric and anatomical guidance during joint training.

For the patient-structure and beam-mask tasks, supervision is implemented using a combination of Dice loss and cross-entropy loss, computed independently for each output channel and then averaged:

$\mathcal{L=}\frac{1}{C}\sum_{c=1}^{C} (\mathcal{L}_{dice}^{\left( c \right)}+\mathcal{L}_{ce}^{\left( c \right)})$ (23)

where $C$denotes the number of output channels for the corresponding auxiliary task (e.g., $C=7$ for anatomical structures and $C=6$ for beam masks). Optional class reweighting is applied to the patient-structure task to account for anatomical imbalance.

The center-distance task is supervised using a voxel-wise mean squared error between the predicted and target continuous distance-field representations.

These auxiliary losses are not intended to be optimized as standalone tasks, but to introduce structured geometric and anatomical supervision that interacts with dose prediction during joint training.

### D.2.6 PTV dose-variance regularization

To discourage unrealistic high-frequency fluctuations within the target, a lightweight regularizer penalizes dose variance inside the PTV:

$\mathcal{L}_{ptv\text{-}var}=\frac{1}{\mid\Omega_{ptv}\mid}\sum_{i\in\Omega_{ptv}} (\hat{D}_{i}-\mu_{ptv})^{2},\mu_{ptv}=\frac{1}{\mid\Omega_{ptv}\mid}\sum_{i\in\Omega_{ptv}} \hat{D}_{i}$ (24)

This term is applied with a small weight $\lambda_{ptv}=0.05$and the same warm-up schedule used for auxiliary tasks. Its role is purely stabilizing and it is deliberately kept minor relative to the main dose objective, ensuring it does not dominate PTV-related evaluation metrics.

## D.3 Training procedure and hyperparameters

### D.3.1 Grid search strategy for hyperparameter selection

To determine a stable and reproducible training configuration, a constrained grid search was conducted over a limited set of global scaling hyperparameters. The goal of this search was not to exhaustively optimize all tunable parameters, but to identify a reasonable operating regime that yields stable convergence under the proposed auxiliary-supervised joint optimization framework.

Specifically, three hyperparameters were explored, resulting in 27 configurations in total:

**Learning rate multiplier**

$$\text{lr\_mult}\in\left\{ 0.5,\text{ }1.0,\text{ }2.0 \right\}$$

applied to a base learning rate of $1\times{10}^{-3}$. This parameter controls the overall optimization step size and was included due to its dominant influence on convergence stability.

**Patient-structure loss scaling factor**

$$s_{\text{patient}}\in\{0.5,\text{ }1.0,\text{ }2.0\}$$

which uniformly rescales the class-weighted loss used for the anatomical structure prediction task. This factor adjusts the relative contribution of patient-structure supervision without altering class-specific weights.

**Dose-loss component scaling factor**

$$s_{\text{comp}}\in\{0.5,\text{ }1.0,\text{ }2.0\}$$

which uniformly rescales the five constituent terms of the main dose loss (MSE, edge consistency, axial consistency, dose-weighted MSE, and DVH matching). This parameter controls the overall strength of dose supervision while preserving the relative balance among its sub-components.

Other parameters—including task-importance priors, auxiliary loss formulations, and architectural settings—were held fixed during the grid search. This design choice was made to avoid confounding task selection effects with extensive hyperparameter tuning and to keep the search space tractable under available computational resources.

Each configuration was evaluated using 5-fold cross-validation on a subset of 472 training plans. The folds were constructed at the plan level and were disjoint from the validation set used during final model training. For each fold, training was run for a maximum of 15 epochs, with early stopping applied when the validation objective did not improve for two consecutive epochs.

Model selection was based on a validation score (val_metric_mae2), defined as a fixed-weight aggregation of the main dose loss components and used exclusively for hyperparameter selection.

Table S3 summarizes the default hyperparameter values prior to global scaling, including optimizer settings, anatomical class weights, dose-loss component weights, task-importance priors, and the high-dose weighting factor used in the dose-weighted MSE term. These values define the reference configuration from which all grid-search variants were derived.

**Table S3. Baseline hyperparameters before grid search and task-level re-scaling**

| **Parameter Category** | **Parameter Name** | **Default Value** | **Description** |
| --- | --- | --- | --- |
| Optimization Parameters | base_lr | 1.00E-03 | Base learning rate before applying lr_mults |
| Structure Weights (Patient) | wp_pctv | 1.5 | PTV weight |
|  | wp_lungl | 1.2 | Left lung weight |
|  | wp_lungr | 1.2 | Right lung weight |
|  | wp_heart | 1.3 | Heart weight |
|  | wp_cord | 1.5 | Spinal cord weight |
|  | wp_breastr | 1.1 | Contralateral breast weight |
|  | wp_body | 1 | Body weight |
| Dose Loss Component Weights | d_fixed_importance | [3, 0.5, 0.2, 0.5, 0.8] | Relative weights of MSE, Edge, Z, Weighted-MSE, and DVH losses |
| Task-level Loss Weights | t_fixed_importance | [1.2, 0.5, 0.5, 0.2] | Base task weights for dose, patient, beam, and center tasks |

**Table S3.** Baseline hyperparameter settings used as reference values prior to grid search, dynamic task re-weighting, and ablation-specific re-scaling. These parameters were held fixed across experiments unless explicitly modified by the grid-search procedure (Section D.3.1) or by task-configuration rules (Section D.3.4). Structure weights correspond to class-wise coefficients in the patient-structure loss, while fixed importance vectors define relative weighting among loss components and tasks.

### D.3.2 Grid Search Results

The grid search results are summarized in Table S4, reporting validation performance across all 27 hyperparameter combinations. Overall, the observed differences in validation loss across configurations were modest, with most settings yielding comparable performance within the variability introduced by cross-validation folds.

Among the evaluated configurations, the setting with a learning-rate multiplier of 2.0, patient-structure loss scaling $s_{\text{patient}}=0.5$, and dose-loss component scaling $s_{\text{comp}}=0.5$achieved the lowest mean validation loss (3.2137 ± 0.68) across the five folds. While the absolute difference relative to neighboring configurations was small, this setting consistently ranked among the top-performing candidates and showed stable convergence behavior within a similar epoch range.

Importantly, no single configuration exhibited a decisive advantage across all metrics, and several neighboring settings achieved comparable validation losses. Therefore, the selected configuration should be interpreted as a representative and well-performing choice within a relatively flat performance landscape, rather than as a uniquely optimal solution.

Based on this observation, the chosen hyperparameter set was adopted for all subsequent experiments to provide a consistent training baseline. This decision prioritizes comparability across task configurations and avoids introducing additional variability due to configuration-specific fine-tuning.

**Table S4. Grid search results for hyperparameter selection**

| **Configuration ID** | **Min Epoch** | **Max Epoch** | **Mean Epochs** | **Mean Validation Score** | **Std. Dev.** |
| --- | --- | --- | --- | --- | --- |
| T001_lr1.0e-03_sp0.5_sc0.5 | 3 | 9 | 5.2 | 3.4637 | 0.532 |
| T001_lr1.0e-03_sp0.5_sc1.0 | 5 | 14 | 8.8 | 3.255 | 0.664 |
| T001_lr1.0e-03_sp0.5_sc2.0 | 3 | 9 | 5.6 | 3.3744 | 0.269 |
| T001_lr1.0e-03_sp1.0_sc0.5 | 6 | 10 | 6.8 | 3.3849 | 0.569 |
| T001_lr1.0e-03_sp1.0_sc1.0 | 3 | 7 | 5.6 | 3.6021 | 0.799 |
| T001_lr1.0e-03_sp1.0_sc2.0 | 2 | 11 | 4.4 | 3.9479 | 1.037 |
| T001_lr1.0e-03_sp2.0_sc0.5 | 2 | 8 | 4.4 | 3.4531 | 0.544 |
| T001_lr1.0e-03_sp2.0_sc1.0 | 3 | 11 | 6.4 | 3.3314 | 0.62 |
| T001_lr1.0e-03_sp2.0_sc2.0 | 3 | 8 | 5.8 | 3.4619 | 0.74 |
| **T001_lr2.0e-03_sp0.5_sc0.5** | **5** | **11** | **7.4** | **3.2137** | **0.678** |
| T001_lr2.0e-03_sp0.5_sc1.0 | 3 | 8 | 5 | 3.4308 | 0.76 |
| T001_lr2.0e-03_sp0.5_sc2.0 | 2 | 10 | 6.2 | 3.3353 | 1.015 |
| T001_lr2.0e-03_sp1.0_sc0.5 | 4 | 10 | 7 | 3.3662 | 0.683 |
| T001_lr2.0e-03_sp1.0_sc1.0 | 3 | 7 | 5.4 | 3.3191 | 0.591 |
| T001_lr2.0e-03_sp1.0_sc2.0 | 4 | 9 | 6.2 | 3.3151 | 0.685 |
| T001_lr2.0e-03_sp2.0_sc0.5 | 4 | 15 | 6.8 | 3.4339 | 0.38 |
| T001_lr2.0e-03_sp2.0_sc1.0 | 3 | 7 | 4.8 | 3.462 | 0.46 |
| T001_lr2.0e-03_sp2.0_sc2.0 | 5 | 9 | 6 | 3.4728 | 0.752 |
| T001_lr5.0e-04_sp0.5_sc0.5 | 2 | 10 | 5.8 | 3.6768 | 0.771 |
| T001_lr5.0e-04_sp0.5_sc1.0 | 4 | 7 | 6 | 3.6111 | 1.053 |
| T001_lr5.0e-04_sp0.5_sc2.0 | 2 | 11 | 6.2 | 3.5244 | 1.105 |
| T001_lr5.0e-04_sp1.0_sc0.5 | 3 | 10 | 5.4 | 3.4073 | 0.533 |
| T001_lr5.0e-04_sp1.0_sc1.0 | 3 | 11 | 6.8 | 3.6725 | 1.098 |
| T001_lr5.0e-04_sp1.0_sc2.0 | 3 | 11 | 7.6 | 3.4974 | 0.877 |
| T001_lr5.0e-04_sp2.0_sc0.5 | 3 | 10 | 6 | 3.4963 | 0.827 |
| T001_lr5.0e-04_sp2.0_sc1.0 | 4 | 12 | 6.4 | 3.3399 | 0.499 |
| T001_lr5.0e-04_sp2.0_sc2.0 | 4 | 15 | 7.4 | 3.3069 | 0.524 |

**Table S4.** Grid-search results for learning rate multiplier (lr), patient-structure scaling (sp), and dose-loss scaling (sc). Reported values show the minimum, maximum, and mean training epochs to convergence, together with the mean and standard deviation of the validation score across 5-fold cross-validation. The configuration selected for final model training is highlighted in bold.

### D.3.3 Final Hyperparameter Settings

Based on the grid-search results summarized in Table S4, a single hyperparameter configuration was selected by jointly considering mean validation performance, cross-fold stability, and convergence behavior, rather than optimizing a single metric alone. The final hyperparameter values used for all subsequent experiments are listed below.

**Table S5. Final effective hyperparameters used for all experiments**

| **Category** | **Parameter** | **Final value** | **Notes** |
| --- | --- | --- | --- |
| Optimization & Training | Optimizer | Adam | PyTorch default (β1=0.9, β2=0.999) |
|  | Weight decay | 0 | Explicitly set to zero |
|  | Base learning rate | 1.0e−3 | Before scaling |
|  | Learning rate multiplier | 2 | Selected by grid search |
|  | Effective learning rate | 2.0e−3 | base_lr × lr_mult |
|  | Max epochs | 15 | Upper bound per run |
|  | Early stopping patience | 2 | Validation-based |
|  | Batch size | 1 | Plans per batch |
| Loss weighting & task configuration | Dose loss component weights | [3, 0.5, 0.2, 0.5, 0.8] | (MSE, Edge, Z, wMSE, DVH); fixed ratios |
|  | Dose loss scaling | s_comp = 0.5 | Selected by grid search |
|  | Patient loss scaling | s_patient = 0.5 | Selected by grid search |
|  | Task importance prior | [1.2, 0.5, 0.5, 0.2] | (dose, patient, beam, center) |
| Dynamic weighting & warm-up | Stability constant ε | 1e−6 | Used in loss normalization |
|  | Ramp horizon S | 1000 steps | Auxiliary warm-up |
|  | Warm-up ratio γ | 0.2 | Full auxiliary weight at S·γ |
| Dose-specific settings | High-dose weight w_max | 5 | Upper bound in dose-weighted MSE |
|  | DVH dose range | 0–70 Gy | Fixed discretization |
|  | DVH bins | 35 | torch.linspace(0,70,steps=35) |
|  | DVH sigmoid steepness k | 20 | Fixed for numerical stability |

**Table S5.** Final effective hyperparameters used for all task configurations (DS, BS, CS, PS, AS). Only the learning-rate multiplier, patient-loss scaling, and dose-loss scaling were selected via grid search; all other parameters were fixed across experiments to ensure comparability between task configurations.

### D.3.4 Ablation Studies and Hyperparameter Inheritance

Ablation experiments were designed to isolate the effect of task selection under a fixed training recipe. All models shared the same network architecture, loss definitions, and hyperparameters determined on the full-task configuration (AS), unless stated otherwise.

Task ablation was implemented exclusively at the loss level. For a given configuration, auxiliary tasks not included in the model were disabled by setting their corresponding task-loss weights to zero. The remaining task-level prior weights (t_fixed_importance) were then re-normalized over the active tasks to keep the overall loss scale comparable across configurations. No loss terms were redefined, and no task-specific formulations were altered.

Because removing tasks can slightly change the effective optimization dynamics, a limited learning-rate adjustment was allowed for each ablation model. Specifically, three candidates {0.5×, 1×, 2×}·lr* were evaluated on the same validation set, while all other hyperparameters were kept fixed. This adjustment was intended solely to maintain stable convergence rather than to optimize task-specific performance.

After selecting the learning rate using the validation set, each task configuration was independently trained using the three prespecified random seeds and evaluated on the held-out test set. The test set was not used for hyperparameter selection. This protocol ensures that observed performance differences primarily reflect the presence or absence of auxiliary task supervision, rather than differences in model capacity, loss design, or training intensity.

## D.4 Characterization of dataset splits and descriptive consistency checks

This section provides a descriptive characterization of anatomical and dosimetric properties across the training, validation, and test splits. All analyses in this section are conducted at the patient level, unless otherwise stated. The analyses are intended to document dataset composition and observed variability following patient-level splitting, rather than to establish formal equivalence or validate the absence of bias.

### D.4.1 Patient-level anatomical volume characterization across dataset splits

To document the anatomical composition of the training, validation, and test datasets, patient-level volumes of selected structures were compared across splits. Specifically, gross target volume–related PTV volume, heart volume, and left lung volume were evaluated using non-parametric Mann–Whitney U tests for pairwise comparisons between splits.

The analysis was conducted at the patient level, with one aggregated volume measurement per patient. Given the limited number of patients in the validation set, these comparisons are intended as descriptive assessments rather than formal tests of equivalence or balance.

As summarized in Table S6, most pairwise comparisons did not show statistically significant differences. For PTV and heart volumes, statistically significant differences were observed between the training and test sets, with moderate effect sizes. No significant differences were observed for left lung volume across any comparison. These results indicate that while certain anatomical characteristics exhibit variability across splits, no systematic or consistent pattern of divergence was observed across all evaluated structures.

Overall, this analysis serves to characterize the distribution of key anatomical volumes following dataset splitting, rather than to establish strict anatomical matching between subsets.

**Table S6. Patient-level anatomical volume comparisons across dataset splits**

| **Comparison Parameters** | **Comparison Groups** | **P-value (Mann-Whitney U Test) (Exact Significance)** | **Sample Size** | **U Statistic** | **Effect Size (r)** |
| --- | --- | --- | --- | --- | --- |
| PTV Volume(gt) | Train(22) vs Validation(3) | 0.969 | 25 | 32 | -0.02 |
|  | Train(22) vs Test(5) | 0.013 | 27 | 16 | -0.47 |
|  | Validation(3) vs Test(5) | 0.143 | 8 | 2 | -0.58 |
| Heart Volume(gt) | Train(22) vs Validation(3) | 0.177 | 25 | 16 | -0.28 |
|  | Train(22) vs Test(5) | 0.04 | 27 | 22 | -0.40 |
|  | Validation(3) vs Test(5) | 0.571 | 8 | 5 | -0.26 |
| Left-Lung Volume(gt) | Train(22) vs Validation(3) | 0.238 | 25 | 18 | -0.25 |
|  | Train(22) vs Test(5) | 0.509 | 27 | 36 | -0.23 |
|  | Validation(3) vs Test(5) | 0.786 | 8 | 6 | -0.16 |

**Table S6.** Patient-level anatomical volumes were compared across dataset splits using Mann–Whitney U tests. Numbers in parentheses denote the number of patients in each split. Effect sizes (r) are reported for descriptive interpretation.

### D.4.2 Patient-level comparison of planning-related dose metrics across dataset splits

To further characterize potential differences in plan-quality–related targets across the training, validation, and test splits, we compared several commonly used dosimetric endpoints derived from the reference (GT) dose distributions, including PTV $D_{95}$, Heart $D_{\text{mean}}$, Left-Lung $V_{20}$, Left-Lung $D_{\text{mean}}$, and Cord $D_{\text{max}}$.

Because multiple treatment plans were generated for each patient under the controlled perturbation design, the analysis was conducted at the patient level. Specifically, for each patient, all available generated plans passing data integrity checks were included. The number of plans per patient ranged from 122 to 130, reflecting minor plan-level exclusions due to corrupted or incomplete files. For each dosimetric endpoint, metric values were first computed for each plan and then averaged across plans within the same patient, yielding a single aggregated value per patient.

Statistical comparisons between dataset splits were subsequently performed on these per-patient aggregated values using Mann–Whitney U tests. The results are summarized in Table S7.

Given the limited number of patients in the validation and test splits, these analyses are intended to provide a descriptive assessment of dataset composition rather than to establish strict equivalence between splits. Observed differences in certain metrics may reflect patient-level anatomical variability and sample size effects, and should therefore be interpreted with appropriate caution.

As summarized in Table S7, most patient-level comparisons of aggregated GT dosimetric metrics across the training, validation, and test splits did not show statistically significant differences under the Mann–Whitney U test. For metrics such as PTV $D_{95}$, Heart $D_{\text{mean}}$, and Left-Lung dose indicators ($V_{20}$ and $D_{\text{mean}}$), p-values were generally above the 0.05 threshold, with small to moderate effect sizes.

A small number of comparisons, most notably those involving Cord $D_{\text{max}}$, yielded nominal statistical significance in pairwise tests. However, these results were observed in the context of very limited sample sizes in the validation and test cohorts, and were accompanied by relatively large effect size estimates that may be sensitive to individual patient characteristics.

Overall, the results in Table S7 suggest that, at the patient-aggregated level, major plan-quality–related dose metrics exhibit broadly comparable distributions across dataset splits, while also highlighting that isolated differences may arise due to patient-level variability and sample size effects. These findings are therefore interpreted as descriptive rather than confirmatory, and are used to contextualize the dataset composition rather than to assert strict equivalence between splits.

**Table S7. Patient-level comparison of dosimetric plan metrics across dataset splits**

| **Comparison Parameters** | **Comparison Groups** | **P-value (Mann-Whitney U Test) (Exact Significance)** | **Sample Size** | **U Statistic** | **Effect Size (r)** |
| --- | --- | --- | --- | --- | --- |
| PTV D95(gt) | Train(22) vs Validation(3) | 0.497 | 25 | 24 | -0.15 |
|  | Train(22) vs Test(5) | 0.314 | 27 | 38 | -0.20 |
|  | Validation(3) vs Test(5) | 0.786 | 8 | 6.5 | -0.11 |
| Heart Dmean(gt) | Train(22) vs Validation(3) | 0.311 | 25 | 20 | -0.22 |
|  | Train(22) vs Test(5) | 0.564 | 27 | 45 | -0.12 |
|  | Validation(3) vs Test(5) | 0.571 | 8 | 5 | -0.26 |
| Left-Lung V20(gt) | Train(22) vs Validation(3) | 0.723 | 25 | 28 | -0.08 |
|  | Train(22) vs Test(5) | 0.284 | 27 | 37 | -0.22 |
|  | Validation(3) vs Test(5) | 1 | 8 | 7 | -0.05 |
| Left-Lung Dmean(gt) | Train(22) vs Validation(3) | 0.273 | 25 | 19 | -0.23 |
|  | Train(22) vs Test(5) | 0.086 | 27 | 27 | -0.34 |
|  | Validation(3) vs Test(5) | 1 | 8 | 7 | -0.05 |
| Cord Dmax(gt) | Train(22) vs Validation(3) | 0.036 | 25 | 8 | -0.42 |
|  | Train(22) vs Test(5) | 0.09 | 27 | 28 | -0.32 |
|  | Validation(3) vs Test(5) | 0.036 | 8 | 0 | -0.79 |

**Table S7.** Mann–Whitney U test results for patient-level comparisons of aggregated GT dosimetric metrics across dataset splits. Values in parentheses following each split name denote the number of patients included (Train: 22, Validation: 3, Test: 5). For each patient, metrics were computed for all available generated plans (122–130 per patient after integrity filtering) and averaged to obtain a single per-patient value used for statistical testing. Effect size is reported as $r$.

# Supplement E. Extended quantitative results

This supplement provides extended numerical results supporting the main text (Results Section 3.1–3.4). Unless otherwise specified, all plan-level metrics were computed on 650 held-out test plans and averaged across three random seeds before cohort-level summarization. Reported confidence intervals (CIs) are two-sided 95% CIs. Abbreviations: DS, single-task baseline; BS, beam-geometry auxiliary task; CS, center auxiliary task; PS, patient-structure auxiliary task; AS, all auxiliary tasks.

## E.1 Extended paired statistical comparisons for voxel-wise errors (MAE/RMSE)

The main text reports overall voxel-wise MAE and RMSE across task configurations (Table 1). Here we provide detailed paired statistical comparisons between each auxiliary-task model and the baseline DS to assess whether observed differences reflect systematic plan-level shifts. In addition to Wilcoxon signed-rank p-values, we report effect sizes and the proportion of negative ranks to aid practical interpretation.

**Table S8. Paired comparison of voxel-wise MAE/RMSE between each model and the single-task baseline (DS).**

| **Pair (model vs ds)** | **Metric** | **Mean Δ (relative%) (95% CI)** | **p (Wilcoxon)** | **Cohen’s d (95% CI)** | **Negative ranks (%)** |
| --- | --- | --- | --- | --- | --- |
| BS-DS | MAE | 0.40(0.08,0.84) | <0.001 | 0.06(-0.01,0.14) | 78.15 |
| BS-DS | RMSE | 0.20(-0.75,0.79) | <0.001 | 0.00(-0.08,0.08) | 76.15 |
| CS-DS | MAE | 2.09(1.96,2.21) | <0.001 | 1.33(1.22,1.43) | 11.08 |
| CS-DS | RMSE | 3.93(3.65,4.21) | <0.001 | 1.08(0.98,1.17) | 19.23 |
| PS-DS | MAE | 3.54(3.26,3.82) | <0.001 | 0.98(0.89,1.07) | 20.92 |
| PS-DS | RMSE | 5.52(5.02,6.02) | <0.001 | 0.85(0.76,0.94) | 20.92 |
| AS-DS | MAE | 2.67(2.55,2.80) | <0.001 | 1.64(1.53,1.76) | 9.23 |
| AS-DS | RMSE | 3.65(3.38,3.91) | <0.001 | 1.05(0.95,1.14) | 20.15 |

**Table S8.** Mean Δ is reported as the relative percentage difference in error (model − DS) with 95% confidence intervals. Two-sided Wilcoxon signed-rank tests were used for paired comparisons across test plans. Cohen’s d is reported to quantify standardized effect size (0.2 small, 0.5 medium, 0.8 large). Negative ranks (%) indicate the proportion of plans for which the model exhibits lower error than DS (i.e., improvement relative to DS). This table complements Table 1 in the main text and supports conclusions regarding whether auxiliary task designs introduce systematic changes in global voxel-wise errors.

## E.2 Extended structure-level bias in clinical dose metrics

Voxel-wise errors may not fully reflect clinically relevant behavior. The main text highlights a subset of key target and OAR endpoints (Table 2 and Figure S2). Here we provide the full structure-level bias profile across PTV and major OAR metrics for all task configurations.

**Table S9.** **PTV-related clinical dose metrics — Mean bias (Prediction − GT)**

| **Metric** | **GT** | **DS** | **BS** | **CS** | **PS** | **AS** |
| --- | --- | --- | --- | --- | --- | --- |
| PTV D95 (Gy) | 45.35 | -1.73 | **-0.67** | -1.89 | -2.38 | -1.63 |
| PTV Dmean (Gy) | 45.77 | **0.04** | 1.21 | 0.3 | -0.54 | 0.15 |
| PTV CI | 0.42 | -0.17 | **-0.03** | -0.12 | -0.28 | -0.19 |
| PTV HI | 1.55 | **6.48** | 7.05 | 8.03 | 6.8 | 6.7 |

**Table S9.** Mean bias (Prediction − GT) for PTV-related clinical dose metrics. Ground truth (GT) values and model bias means are reported for PTV endpoints, including D95 (coverage), Dmean, conformity index (CI), and heterogeneity index (HI). Mean bias values are computed across 650 test plans after averaging predictions over three random seeds; negative values indicate systematic underestimation relative to GT. Values are shown to two decimal places for readability. Highlighted (bold) entries denote the minimum mean bias within each row based on full-precision calculations.

**Table S10. PTV-related clinical dose metrics — 95% confidence intervals**

| **Metric** | **GT (95% CI)** | **DS (95% CI)** | **BS (95% CI)** | **CS (95% CI)** | **PS (95% CI)** | **AS (95% CI)** |
| --- | --- | --- | --- | --- | --- | --- |
| PTV D95 (Gy) | (44.73,45.96) | (-2.34,-1.07) | **(-1.31,-0.03)** | (-2.52,-1.25) | (-3.01,-1.74) | (-2.27,-0.99) |
| PTV Dmean (Gy) | (45.15,46.40) | **(-0.60,0.69)** | (0.56,1.86) | (-0.35,0.94) | (-1.19,0.11) | (-0.50,0.80) |
| PTV CI | (0.41,0.44) | (-0.19,-0.16) | **(-0.05,-0.01)** | (-0.14,-0.10) | (-0.30,-0.27) | (-0.21,-0.18) |
| PTV HI(%) | (1.49,1.61) | **(6.33,6.63)** | (6.92,7.18) | (7.89,8.18) | (6.66,6.94) | (6.56,6.84) |

**Table S10.** Two-sided 95% confidence intervals (CI) corresponding to the mean bias values reported in Table S9 for PTV-related clinical dose metrics. Confidence intervals are computed across 650 test plans after averaging predictions over three random seeds. Bias is defined as Prediction − GT. This table complements Table S9 by reporting uncertainty estimates separately from mean values.

**Table S11. OAR-related clinical dose metrics — Mean bias (Prediction − GT)**

| **Metric** | **GT Mean** | **DS Mean** | **BS Mean** | **CS Mean** | **PS Mean** | **AS Mean** |
| --- | --- | --- | --- | --- | --- | --- |
| Heart Dmean(Gy) | 9.21 | -1.73 | **-0.95** | -1.4 | -1.64 | -1.25 |
| Heart V5(fraction) | 0.49 | -0.1 | -0.07 | -0.07 | -0.09 | **-0.06** |
| Cord Max(Gy) | 26.3 | -6.7 | **-5.01** | -6.28 | -7.04 | -5.82 |
| LungL Dmean(Gy) | 16.94 | -2.69 | **-1.68** | -2.29 | -2.89 | -2.22 |
| LungL V5(fraction) | 0.58 | -0.08 | -0.07 | -0.07 | -0.08 | **-0.06** |
| LungL V20(fraction) | 0.37 | -0.06 | **-0.03** | -0.05 | -0.06 | -0.04 |
| LungR V5(fraction) | 0.06 | -0.02 | **-0.01** | -0.01 | -0.02 | -0.02 |
| BreastR Dmean(Gy) | 3.54 | **0.49** | 1.23 | 0.94 | 0.97 | 0.83 |
| Body Dmean(Gy) | 8.8 | -0.58 | **-0.22** | -0.55 | -0.75 | -0.46 |

**Table S11.** Mean bias (Prediction − GT) for major organs-at-risk (OAR) clinical dose metrics. Ground truth (GT) values and model bias means are reported for representative OAR endpoints, including mean dose and volume-at-dose metrics for critical organs (e.g., Heart, Spinal cord, Lungs, Breast, and Body). Mean bias values are computed across 650 test plans after averaging predictions over three random seeds; negative values indicate systematic underestimation relative to GT. Values are shown to two decimal places for readability. Highlighted (bold) entries denote the smallest mean bias within each row based on full-precision calculations.

**Table S12. OAR-related clinical dose metrics — 95% confidence intervals**

| **Metric** | **GT 95% CI** | **DS 95% CI** | **BS 95% CI** | **CS 95% CI** | **PS 95% CI** | **AS 95% CI** |
| --- | --- | --- | --- | --- | --- | --- |
| Heart Dmean(Gy) | (9.00,9.41) | (-1.92,-1.53) | **(-1.13,-0.78)** | (-1.58,-1.21) | (-1.83,-1.46) | (-1.43,-1.08) |
| Heart V5(fraction) | (0.48,0.50) | (-0.11,-0.08) | (-0.08,-0.06) | (-0.08,-0.06) | (-0.10,-0.08) | **(-0.08,-0.05)** |
| Cord Max(Gy) | (25.69,26.91) | (-7.23,-6.16) | **(-5.58,-4.44)** | (-6.82,-5.73) | (-7.59,-6.49) | (-6.38,-5.26) |
| LungL Dmean(Gy) | (16.83,17.06) | (-2.78,-2.60) | **(-1.77,-1.59)** | (-2.39,-2.20) | (-2.98,-2.80) | (-2.30,-2.13) |
| LungL V5(fraction) | (0.57,0.59) | (-0.09,-0.07) | (-0.08,-0.06) | (-0.08,-0.06) | (-0.10,-0.07) | **(-0.07,-0.05)** |
| LungL V20(fraction) | (0.36,0.37) | (-0.06,-0.05) | **(-0.04,-0.03)** | (-0.06,-0.05) | (-0.06,-0.05) | (-0.05,-0.04) |
| LungR V5(fraction) | (0.06,0.07) | (-0.03,-0.02) | **(-0.02,-0.01)** | (-0.02,-0.01) | (-0.03,-0.02) | (-0.02,-0.02) |
| BreastR Dmean(Gy) | (3.44,3.64) | **(0.38,0.60)** | (1.10,1.37) | (0.83,1.06) | (0.85,1.10) | (0.71,0.95) |
| Body Dmean(Gy) | (8.73,8.88) | (-0.69,-0.48) | **(-0.33,-0.10)** | (-0.66,-0.45) | (-0.86,-0.64) | (-0.57,-0.35) |

**Table S12.** Two-sided 95% confidence intervals (CI) corresponding to the mean bias values reported in Table S11 for major OAR-related clinical dose metrics. Confidence intervals are computed across 650 test plans after averaging predictions over three random seeds. Bias is defined as Prediction − GT. This table provides uncertainty estimates complementary to the mean bias summaries in Table S11 and supports the trends discussed in Table 2 and visualized in Fig. S1.

**Figure S2. Bias of clinical dose metrics across task configurations (Prediction − GT).** (a) PTV-related metrics (D95, Dmean, CI, HI). (b) Selected OAR metrics, including heart, spinal cord, lungs, breast, and body. Negative values denote systematic underestimation. For metrics with very small absolute bias, numerical values are annotated for clarity.

## E.3 Full-dose-threshold spatial overlap (DSC) in isodose regions

As an additional spatial evaluation, Dice similarity coefficients (DSC) were computed for thresholded isodose regions across the full evaluated dose-threshold range (5–50 Gy). Results for the Body and PTV regions are reported in Tables S13 and S14, respectively.

**Table S13. Spatial overlap (DSC) across all isodose thresholds in Body regions (thresholds of 5, 10, 20, 30, 40, and 50 Gy).**

| **Body isodose level** | **DSC_DS mean±SD** | **DSC_BS mean±SD** | **DSC_CS mean±SD** | **DSC_PS mean±SD** | **DSC_AS mean±SD** |
| --- | --- | --- | --- | --- | --- |
| D5Gy | **0.84 ± 0.02** | 0.84 ± 0.02 | 0.84 ± 0.02 | 0.84 ± 0.02 | 0.84 ± 0.02 |
| D10Gy | **0.86 ± 0.02** | 0.86 ± 0.02 | 0.85 ± 0.02 | 0.85 ± 0.02 | 0.86 ± 0.02 |
| D20Gy | 0.87 ± 0.02 | **0.87 ± 0.03** | 0.86 ± 0.02 | 0.86 ± 0.02 | 0.86 ± 0.02 |
| D30Gy | 0.81 ± 0.11 | **0.81 ± 0.12** | 0.80 ± 0.10 | 0.80 ± 0.10 | 0.80 ± 0.11 |
| D40Gy | 0.62 ± 0.32 | **0.65 ± 0.33** | 0.61 ± 0.32 | 0.60 ± 0.31 | 0.62 ± 0.32 |
| D50Gy | 0.02 ± 0.01 | **0.09 ± 0.05** | 0.05 ± 0.03 | 0.01 ± 0.01 | 0.03 ± 0.02 |

**Table S13.** This table reports Dice similarity coefficient (DSC)–based spatial overlap of thresholded isodose regions for the Body ROI across dose thresholds from D5 to D50 Gy. For a given threshold $T$(Gy), the isodose region is defined as the voxel set $\left\{ v\mid D(v)\geq T \right\}$ and is restricted to the corresponding ROI. This table complements Section 3.3 by providing the full threshold spectrum beyond the high-dose subset highlighted in the main text. Values are shown to two decimal places for readability. Highlighted (bold) entries denote the biggest mean value within each row based on full-precision calculations.

**Table S14. Spatial overlap (DSC) across all isodose thresholds in PTV regions (thresholds of 5, 10, 20, 30, 40, and 50 Gy).**

| **PTV isodose level** | **DSC_DS**  **mean±SD** | **DSC_BS**  **mean±SD** | **DSC_CS**  **mean±SD** | **DSC_PS**  **mean±SD** | **DSC_AS mean±SD** |
| --- | --- | --- | --- | --- | --- |
| D5Gy | 1.00 ± 0.00 | 1.00 ± 0.00 | 1.00 ± 0.00 | 1.00 ± 0.00 | 1.00 ± 0.00 |
| D10Gy | 1.00 ± 0.00 | 1.00 ± 0.00 | 1.00 ± 0.00 | 1.00 ± 0.00 | 1.00 ± 0.00 |
| D20Gy | 1.00 ± 0.00 | 1.00 ± 0.00 | 1.00 ± 0.00 | 1.00 ± 0.00 | 1.00 ± 0.00 |
| D30Gy | 0.98 ± 0.03 | 0.98 ± 0.03 | 0.98 ± 0.03 | 0.98 ± 0.03 | 0.98 ± 0.03 |
| D40Gy | 0.78 ± 0.40 | **0.79 ± 0.41** | 0.78 ± 0.40 | 0.78 ± 0.40 | 0.79 ± 0.40 |
| D50Gy | 0.02 ± 0.01 | **0.10 ± 0.06** | 0.06 ± 0.04 | 0.01 ± 0.01 | 0.04 ± 0.02 |

**Table S14.** This table shows the Dice similarity coefficient (DSC) values for spatial overlap of thresholded isodose regions within the PTV region of interest (ROI) at various dose levels from D5 to D50 Gy. The isodose region at a given threshold T (Gy) is defined as the voxel set {v | D(v) ≥ T} and is limited to the specified ROI. This table extends the analysis in Section 3.3 by including the full range of dose thresholds, beyond the high-dose range (D40–D50) discussed in the main text. All values are rounded to two decimal places for readability, and the largest mean value in each row is marked in bold, calculated with full-precision values.

## E.4 Quartile stratification of sub-task loss vs main-task error

To further explore the association between auxiliary-task performance and dose-prediction error, we performed two descriptive plan-level analyses. First, we report Spearman rank correlations between each sub-task loss and voxel-wise MAE/RMSE. Second, we present a quartile-based stratification comparing prediction errors between low-loss (Q1) and high-loss (Q4) groups for interpretability. For consistency with the main analysis, each association is evaluated within the corresponding task-enabled configuration only (beam loss in BS, center loss in CS, and patient loss in PS).

**Table S15. Spearman rank correlation between sub-task losses and dose prediction errors**

| **Sub-Task** | **Correlation with MAE** | **P-value (MAE)** | **Correlation with RMSE** | **P-value (RMSE)** |
| --- | --- | --- | --- | --- |
| beam_loss | 0.29 | <0.001 | 0.27 | <0.001 |
| center_loss | -0.29 | <0.001 | -0.24 | <0.001 |
| patient_loss | 0.16 | <0.001 | 0.10 | <0.001 |

**Table S15.** Spearman rank correlations (ρ) between sub-task losses and voxel-wise MAE/RMSE at the plan level. Each correlation was computed within the corresponding task-enabled configuration only (beam loss in BS, center loss in CS, and patient loss in PS). For each plan, MAE/RMSE and the corresponding sub-task loss were first computed independently for each random seed and then averaged across three seeds to obtain a single plan-level value. P-values are two-sided and are reported for descriptive purposes; these associations are not intended to imply causal effects.

**Table S16. Quartile-stratified differences in voxel-wise MAE/RMSE between low-loss (Q1) and high-loss (Q4) groups for each sub-task loss.**

| **Sub-task** | **Loss (Q1) mean ± SD** | **Loss (Q4) mean ± SD** | **MAE (Q1) mean ± SD** | **MAE (Q4) mean ± SD** | **ΔMAE (Q4−Q1)** | **p_MAE** | **RMSE (Q1) mean ± SD** | **RMSE (Q4) mean ± SD** | **ΔRMSE (Q4−Q1)** | **p_RMSE** |
| --- | --- | --- | --- | --- | --- | --- | --- | --- | --- | --- |
| **beam** | 2.27±0.02 | 2.38±0.02 | 2.68±0.24 | 3.85±0.93 | 1.17 | <0.001 | 4.97±0.96 | 6.45±1.46 | 1.48 | <0.001 |
| **center** | 0.07±0.00 | 0.11±0.01 | 3.74±0.61 | 2.86±0.15 | -0.88 | <0.001 | 6.29±0.93 | 5.33±0.90 | -0.96 | <0.001 |
| **patient** | 3.21±0.00 | 3.33±0.00 | 2.93±0.15 | 3.95±0.09 | 1.02 | <0.001 | 5.53±0.95 | 6.58±0.14 | 1.05 | <0.001 |

**Table S16.** Quartile-stratified comparison of main-task prediction errors between plans in the lowest (Q1) and highest (Q4) quartiles of each sub-task loss(beam_loss, center_loss, patient_loss). Quartiles were defined separately for each sub-task loss using its empirical plan-level distribution. For each plan, voxel-wise MAE and RMSE were first averaged across three random seeds, and group summaries are reported as mean ± SD for Q1 and Q4. The between-group difference is reported as $\Delta=Q4-Q1$, where positive values indicate higher prediction error in the high-loss group. Group differences were assessed using two-sided Mann–Whitney U tests. This table complements the correlation analysis in Table 4 and provides an interpretable stratified view of how sub-task loss levels relate to main-task prediction error.

## E.5 Additional analyses of optimization coupling, auxiliary-input dependence, and robustness

Additional analyses were performed to assess whether the observed auxiliary-task effects were robust across random initialization, dependent on the primary dose-loss formulation, and related to the use of predicted-dose information by the auxiliary tasks. All relevant experiments were evaluated across three random seeds (42, 123, and 456).

To examine sensitivity to the primary dose objective, the original composite dose loss was replaced with voxel-wise mean squared error (MSE), while the task configurations, data splits, network architectures, and evaluation procedure were otherwise retained. Seed-specific performance across the five model configurations is reported separately for the composite-loss and MSE-only settings in Tables S17 and S18, respectively. For dosimetric endpoints in the MSE-only analysis, endpoint error was calculated as the case-level absolute difference between the predicted and ground-truth quality indices, $\mid QI_{\mathrm{pred}}-QI_{\mathrm{gt}}\mid$, and then averaged across cases.To directly assess the effect of auxiliary supervision relative to the corresponding dose-only baseline (DS), seed-specific paired differences were calculated as

$$\Delta=E_{\mathrm{aux}}-E_{\mathrm{DS}},$$

where $E$ denotes the absolute endpoint prediction error for the same random seed. Negative values therefore indicate lower error than DS, whereas positive values indicate higher error. The seed-specific paired effects are reported for the composite-loss and MSE-only settings in Tables S19 and S20, respectively. Table S21 summarizes the corresponding cross-loss comparison, including the cross-seed mean paired effect and the number of seeds showing improvement.

**Table S17. Seed-specific performance across model variants under composite-loss training**

| **Model** | **Metric** | **Seed 42** | **Seed 123** | **Seed 456** | **Multi-seed mean ± SD** |
| --- | --- | --- | --- | --- | --- |
| **AS** | MAE | 2.82 | 3.01 | 3.26 | 3.03 ± 0.22 |
|  | RMSE | 4.86 | 5.18 | 5.86 | 5.30 ± 0.51 |
|  | Cord_max | 23.82 | 20.27 | 17.37 | 20.48 ± 3.23 |
|  | Heart_mean | 9.44 | 7.39 | 7.03 | 7.95 ± 1.30 |
|  | Heart_v5 | 0.51 | 0.38 | 0.39 | 0.43 ± 0.07 |
|  | Breast_R_mean | 5.49 | 4.08 | 3.54 | 4.37 ± 1.01 |
|  | PTV_D95 | 46.59 | 42.3 | 42.27 | 43.72 ± 2.49 |
|  | PTV_mean | 48.48 | 44.41 | 44.86 | 45.92 ± 2.23 |
|  | PTV_CI | 0.64 | 0.01 | 0.05 | 0.23 ± 0.35 |
|  | PTV_HI | 7.29 | 7.68 | 9.79 | 8.25 ± 1.35 |
|  | Body_mean | 9.36 | 8.23 | 7.44 | 8.34 ± 0.97 |
|  | Lung_L_v5 | 0.56 | 0.51 | 0.49 | 0.52 ± 0.03 |
|  | Lung_L_v20 | 0.34 | 0.33 | 0.3 | 0.32 ± 0.02 |
|  | Lung_L_mean | 16.26 | 14.44 | 13.48 | 14.73 ± 1.41 |
|  | Lung_R_v5 | 0.07 | 0.03 | 0.03 | 0.04 ± 0.02 |
| **BS** | MAE | 2.82 | 3.02 | 3.04 | 2.96 ± 0.12 |
|  | RMSE | 4.92 | 5.22 | 5.45 | 5.19 ± 0.27 |
|  | Cord_max | 24.13 | 19.48 | 20.26 | 21.29 ± 2.49 |
|  | Heart_mean | 9.44 | 7.18 | 8.15 | 8.26 ± 1.13 |
|  | Heart_v5 | 0.51 | 0.37 | 0.39 | 0.42 ± 0.08 |
|  | Breast_R_mean | 5.48 | 4.13 | 4.71 | 4.77 ± 0.68 |
|  | PTV_D95 | 46.76 | 42.01 | 45.28 | 44.68 ± 2.43 |
|  | PTV_mean | 48.63 | 44.09 | 48.21 | 46.98 ± 2.51 |
|  | PTV_CI | 0.65 | 0 | 0.52 | 0.39 ± 0.34 |
|  | PTV_HI | 7.11 | 7.52 | 11.16 | 8.60 ± 2.23 |
|  | Body_mean | 9.35 | 7.99 | 8.42 | 8.59 ± 0.69 |
|  | Lung_L_v5 | 0.55 | 0.49 | 0.5 | 0.51 ± 0.03 |
|  | Lung_L_v20 | 0.34 | 0.32 | 0.35 | 0.34 ± 0.02 |
|  | Lung_L_mean | 16.17 | 13.91 | 15.7 | 15.26 ± 1.19 |
|  | Lung_R_v5 | 0.07 | 0.03 | 0.04 | 0.05 ± 0.02 |
| **CS** | MAE | 2.83 | 3.11 | 3.09 | 3.01 ± 0.16 |
|  | RMSE | 4.9 | 5.42 | 5.61 | 5.31 ± 0.37 |
|  | Cord_max | 23.85 | 18.43 | 17.8 | 20.03 ± 3.33 |
|  | Heart_mean | 9.81 | 6.82 | 6.81 | 7.81 ± 1.73 |
|  | Heart_v5 | 0.5 | 0.35 | 0.42 | 0.42 ± 0.08 |
|  | Breast_R_mean | 5.35 | 3.92 | 4.18 | 4.48 ± 0.76 |
|  | PTV_D95 | 45.67 | 40.85 | 43.86 | 43.46 ± 2.43 |
|  | PTV_mean | 48.28 | 43.14 | 46.79 | 46.07 ± 2.65 |
|  | PTV_CI | 0.58 | 0 | 0.32 | 0.30 ± 0.29 |
|  | PTV_HI | 9.29 | 8.22 | 11.25 | 9.59 ± 1.53 |
|  | Body_mean | 9.2 | 7.67 | 7.89 | 8.25 ± 0.83 |
|  | Lung_L_v5 | 0.54 | 0.49 | 0.51 | 0.51 ± 0.03 |
|  | Lung_L_v20 | 0.35 | 0.31 | 0.29 | 0.32 ± 0.03 |
|  | Lung_L_mean | 16.49 | 13.38 | 14.08 | 14.65 ± 1.63 |
|  | Lung_R_v5 | 0.08 | 0.03 | 0.05 | 0.05 ± 0.02 |
| **PS** | MAE | 2.82 | 3.09 | 3.25 | 3.05 ± 0.22 |
|  | RMSE | 4.89 | 5.36 | 5.82 | 5.36 ± 0.46 |
|  | Cord_max | 21.82 | 18.58 | 17.39 | 19.26 ± 2.29 |
|  | Heart_mean | 8.84 | 6.83 | 7.04 | 7.57 ± 1.10 |
|  | Heart_v5 | 0.46 | 0.35 | 0.4 | 0.40 ± 0.06 |
|  | Breast_R_mean | 5.77 | 3.9 | 3.87 | 4.51 ± 1.09 |
|  | PTV_D95 | 45.33 | 41.3 | 42.29 | 42.97 ± 2.10 |
|  | PTV_mean | 47.4 | 43.55 | 44.75 | 45.23 ± 1.97 |
|  | PTV_CI | 0.38 | 0 | 0.03 | 0.14 ± 0.21 |
|  | PTV_HI | 7.58 | 8.18 | 9.3 | 8.35 ± 0.88 |
|  | Body_mean | 8.88 | 7.77 | 7.5 | 8.05 ± 0.73 |
|  | Lung_L_v5 | 0.53 | 0.49 | 0.48 | 0.50 ± 0.02 |
|  | Lung_L_v20 | 0.33 | 0.31 | 0.3 | 0.31 ± 0.02 |
|  | Lung_L_mean | 15.38 | 13.54 | 13.25 | 14.06 ± 1.16 |
|  | Lung_R_v5 | 0.06 | 0.03 | 0.03 | 0.04 ± 0.02 |
| **DS** | MAE | 2.78 | 3.13 | 2.94 | 2.95 ± 0.17 |
|  | RMSE | 4.83 | 5.44 | 5.31 | 5.19 ± 0.33 |
|  | Cord_max | 22.71 | 18.39 | 17.72 | 19.61 ± 2.71 |
|  | Heart_mean | 8.43 | 6.66 | 7.36 | 7.48 ± 0.89 |
|  | Heart_v5 | 0.44 | 0.34 | 0.4 | 0.39 ± 0.05 |
|  | Breast_R_mean | 5.3 | 3.98 | 2.81 | 4.03 ± 1.25 |
|  | PTV_D95 | 45.63 | 40.63 | 44.66 | 43.64 ± 2.65 |
|  | PTV_mean | 47.74 | 42.96 | 46.75 | 45.82 ± 2.52 |
|  | PTV_CI | 0.52 | 0 | 0.23 | 0.25 ± 0.26 |
|  | PTV_HI | 7.53 | 8.38 | 8.18 | 8.03 ± 0.45 |
|  | Body_mean | 8.97 | 7.68 | 8.01 | 8.22 ± 0.67 |
|  | Lung_L_v5 | 0.53 | 0.49 | 0.49 | 0.50 ± 0.02 |
|  | Lung_L_v20 | 0.32 | 0.3 | 0.31 | 0.31 ± 0.01 |
|  | Lung_L_mean | 15.22 | 13.24 | 14.31 | 14.26 ± 0.99 |
|  | Lung_R_v5 | 0.07 | 0.03 | 0.02 | 0.04 ± 0.03 |

**Table note.** Values are reported for three independent random-seed runs (42, 123, and 456). Multi-seed results are presented as mean ± standard deviation (SD) across the three runs. All numerical values are rounded to two decimal places. AS, BS, CS, PS, and DS denote the five model variants defined in the main text.

**Table S18. Seed-specific endpoint performance across model variants under MSE-only training**

| **Model** | **Metric** | **Seed 42** | **Seed 123** | **Seed 456** | **Multi-seed mean ± SD** |
| --- | --- | --- | --- | --- | --- |
| **AS** | MAE | 2.78 | 2.73 | 2.73 | 2.75 ± 0.03 |
|  | RMSE | 4.83 | 4.87 | 4.93 | 4.88 ± 0.05 |
|  | Cord_max | 5.17 | 5.43 | 6.58 | 5.73 ± 0.75 |
|  | Heart_mean | 2.08 | 1.93 | 2.22 | 2.08 ± 0.15 |
|  | Heart_v5 | 0.15 | 0.12 | 0.12 | 0.13 ± 0.02 |
|  | Breast_R_mean | 1.85 | 1.18 | 0.98 | 1.34 ± 0.46 |
|  | PTV_D95 | 6.89 | 7.69 | 7.1 | 7.23 ± 0.41 |
|  | PTV_mean | 5.46 | 6.17 | 5.26 | 5.63 ± 0.48 |
|  | PTV_CI | 0.23 | 0.31 | 0.15 | 0.23 ± 0.08 |
|  | PTV_HI | 11.76 | 10.47 | 12.97 | 11.73 ± 1.25 |
|  | Body_mean | 0.9 | 1.01 | 1.15 | 1.02 ± 0.12 |
|  | Lung_L_v5 | 0.09 | 0.1 | 0.09 | 0.09 ± 0.00 |
|  | Lung_L_v20 | 0.04 | 0.05 | 0.06 | 0.05 ± 0.01 |
|  | Lung_L_mean | 1.26 | 1.67 | 2.27 | 1.73 ± 0.51 |
|  | Lung_R_v5 | 0.04 | 0.04 | 0.04 | 0.04 ± 0.00 |
| **BS** | MAE | 2.77 | 2.95 | 3 | 2.90 ± 0.12 |
|  | RMSE | 4.82 | 5.22 | 5.07 | 5.04 ± 0.21 |
|  | Cord_max | 6.06 | 8 | 6.4 | 6.82 ± 1.04 |
|  | Heart_mean | 2.48 | 2.09 | 1.66 | 2.08 ± 0.41 |
|  | Heart_v5 | 0.17 | 0.12 | 0.12 | 0.14 ± 0.03 |
|  | Breast_R_mean | 1.65 | 0.98 | 1.79 | 1.48 ± 0.43 |
|  | PTV_D95 | 7.39 | 9.78 | 9.2 | 8.79 ± 1.25 |
|  | PTV_mean | 5.81 | 8.06 | 7.81 | 7.23 ± 1.23 |
|  | PTV_CI | 0.27 | 0.41 | 0.41 | 0.36 ± 0.09 |
|  | PTV_HI | 12.07 | 12.05 | 10.71 | 11.61 ± 0.78 |
|  | Body_mean | 0.95 | 1.58 | 1.15 | 1.23 ± 0.32 |
|  | Lung_L_v5 | 0.09 | 0.1 | 0.09 | 0.10 ± 0.01 |
|  | Lung_L_v20 | 0.05 | 0.07 | 0.06 | 0.06 ± 0.01 |
|  | Lung_L_mean | 1.57 | 3.49 | 2.43 | 2.50 ± 0.96 |
|  | Lung_R_v5 | 0.04 | 0.04 | 0.04 | 0.04 ± 0.00 |
| **CS** | MAE | 2.79 | 2.94 | 2.77 | 2.84 ± 0.09 |
|  | RMSE | 4.91 | 5.17 | 4.75 | 4.94 ± 0.21 |
|  | Cord_max | 7.31 | 9.38 | 5.27 | 7.32 ± 2.05 |
|  | Heart_mean | 2.19 | 2.08 | 1.73 | 2.00 ± 0.24 |
|  | Heart_v5 | 0.15 | 0.12 | 0.13 | 0.13 ± 0.01 |
|  | Breast_R_mean | 1.18 | 1 | 1.13 | 1.10 ± 0.09 |
|  | PTV_D95 | 7.96 | 9.48 | 7.43 | 8.29 ± 1.07 |
|  | PTV_mean | 6.36 | 7.73 | 5.84 | 6.65 ± 0.98 |
|  | PTV_CI | 0.34 | 0.41 | 0.24 | 0.33 ± 0.09 |
|  | PTV_HI | 11.13 | 11.81 | 11.32 | 11.42 ± 0.35 |
|  | Body_mean | 1.04 | 1.49 | 0.84 | 1.12 ± 0.33 |
|  | Lung_L_v5 | 0.08 | 0.1 | 0.11 | 0.10 ± 0.01 |
|  | Lung_L_v20 | 0.07 | 0.07 | 0.05 | 0.06 ± 0.01 |
|  | Lung_L_mean | 2.66 | 3.09 | 1.57 | 2.44 ± 0.78 |
|  | Lung_R_v5 | 0.04 | 0.04 | 0.04 | 0.04 ± 0.00 |
| **PS** | MAE | 2.72 | 2.87 | 3.05 | 2.88 ± 0.16 |
|  | RMSE | 4.72 | 4.94 | 5.26 | 4.97 ± 0.27 |
|  | Cord_max | 5.93 | 5.98 | 6.59 | 6.16 ± 0.36 |
|  | Heart_mean | 2.1 | 1.72 | 1.82 | 1.88 ± 0.20 |
|  | Heart_v5 | 0.14 | 0.14 | 0.12 | 0.13 ± 0.01 |
|  | Breast_R_mean | 1.84 | 3.12 | 1.4 | 2.12 ± 0.89 |
|  | PTV_D95 | 7.01 | 6.93 | 10.04 | 7.99 ± 1.77 |
|  | PTV_mean | 5.86 | 5.46 | 7.73 | 6.35 ± 1.21 |
|  | PTV_CI | 0.26 | 0.18 | 0.4 | 0.28 ± 0.11 |
|  | PTV_HI | 9.93 | 11.58 | 15.7 | 12.40 ± 2.97 |
|  | Body_mean | 0.85 | 0.87 | 1.19 | 0.97 ± 0.19 |
|  | Lung_L_v5 | 0.09 | 0.1 | 0.09 | 0.09 ± 0.01 |
|  | Lung_L_v20 | 0.05 | 0.05 | 0.05 | 0.05 ± 0.00 |
|  | Lung_L_mean | 1.58 | 1.61 | 2.08 | 1.75 ± 0.28 |
|  | Lung_R_v5 | 0.04 | 0.04 | 0.04 | 0.04 ± 0.00 |
| **DS** | MAE | 2.7 | 2.86 | 3.1 | 2.89 ± 0.20 |
|  | RMSE | 4.77 | 4.89 | 5.28 | 4.98 ± 0.26 |
|  | Cord_max | 5.62 | 6.1 | 5.92 | 5.88 ± 0.24 |
|  | Heart_mean | 2.21 | 1.58 | 1.67 | 1.82 ± 0.34 |
|  | Heart_v5 | 0.14 | 0.14 | 0.12 | 0.13 ± 0.01 |
|  | Breast_R_mean | 1.38 | 2.39 | 1.65 | 1.81 ± 0.52 |
|  | PTV_D95 | 7.09 | 7.24 | 10.03 | 8.12 ± 1.66 |
|  | PTV_mean | 5.87 | 5.77 | 7.72 | 6.45 ± 1.10 |
|  | PTV_CI | 0.26 | 0.21 | 0.4 | 0.29 ± 0.10 |
|  | PTV_HI | 10.36 | 11.65 | 15.64 | 12.55 ± 2.76 |
|  | Body_mean | 0.86 | 0.88 | 1.09 | 0.95 ± 0.13 |
|  | Lung_L_v5 | 0.09 | 0.1 | 0.1 | 0.10 ± 0.01 |
|  | Lung_L_v20 | 0.05 | 0.05 | 0.05 | 0.05 ± 0.00 |
|  | Lung_L_mean | 1.49 | 1.4 | 1.81 | 1.57 ± 0.21 |
|  | Lung_R_v5 | 0.04 | 0.04 | 0.04 | 0.04 ± 0.00 |

**Table note.** Values are reported for three independent random-seed runs (42, 123, and 456) under MSE-only training. Multi-seed results are presented as mean ± standard deviation (SD) across the three runs. All numerical values are rounded to two decimal places. AS, BS, CS, PS, and DS denote the five model variants defined in the main text.

**Table S19. Seed-specific paired endpoint effects relative to DS under composite-loss training**

| **Metric** | **Comparison** | **Δ42** | **Δ123** | **Δ456** | **Mean Δ ± SDΔ** | **Seeds improved** |
| --- | --- | --- | --- | --- | --- | --- |
| MAE(Gy) | AS vs DS | 0.03 | -0.12 | 0.32 | 0.08 ± 0.23 | 1/3 |
|  | BS vs DS | 0.04 | -0.11 | 0.10 | 0.01 ± 0.11 | 1/3 |
|  | CS vs DS | 0.05 | -0.02 | 0.15 | 0.06 ± 0.09 | 1/3 |
|  | PS vs DS | 0.04 | -0.04 | 0.32 | 0.10 ± 0.19 | 1/3 |
| RMSE(Gy) | AS vs DS | 0.04 | -0.27 | 0.55 | 0.11 ± 0.41 | 1/3 |
|  | BS vs DS | 0.09 | -0.23 | 0.14 | 0.00 ± 0.20 | 1/3 |
|  | CS vs DS | 0.07 | -0.02 | 0.30 | 0.12 ± 0.16 | 1/3 |
|  | PS vs DS | 0.07 | -0.09 | 0.51 | 0.16 ± 0.31 | 1/3 |
| Cord_max | AS vs DS | -0.58 | -0.71 | 0.38 | -0.30 ± 0.60 | 2/3 |
|  | BS vs DS | -0.66 | -0.60 | -0.41 | -0.56 ± 0.13 | **3/3** |
|  | CS vs DS | -0.49 | -0.05 | 0.24 | -0.10 ± 0.37 | 2/3 |
|  | PS vs DS | 0.62 | -0.04 | 0.41 | +0.33 ± 0.33 | 1/3 |
| Heart_mean | AS vs DS | 0.08 | -0.63 | 0.05 | -0.16 ± 0.40 | 1/3 |
|  | BS vs DS | 0.23 | -0.39 | -0.66 | -0.27 ± 0.46 | 2/3 |
|  | CS vs DS | 0.36 | -0.12 | 0.24 | +0.16 ± 0.25 | 1/3 |
|  | PS vs DS | 0.05 | -0.21 | 0.06 | -0.03 ± 0.15 | 1/3 |
| Heart_v5 | AS vs DS | 0.00 | -0.04 | -0.01 | -0.02 ± 0.02 | **3/3** |
|  | BS vs DS | 0.00 | -0.02 | -0.02 | -0.01 ± 0.01 | 2/3 |
|  | CS vs DS | 0.01 | -0.01 | -0.03 | -0.01 ± 0.02 | 2/3 |
|  | PS vs DS | 0.00 | -0.01 | 0.00 | -0.00± 0.00 | 1/3 |
| Breast_R_mean | AS vs DS | 0.18 | -0.04 | 0.07 | +0.07 ± 0.11 | 1/3 |
|  | BS vs DS | 0.16 | 0.07 | 0.54 | +0.26 ± 0.25 | 0/3 |
|  | CS vs DS | 0.04 | -0.02 | 0.02 | +0.01 ± 0.03 | 1/3 |
|  | PS vs DS | 0.43 | 0.03 | 0.14 | +0.20 ± 0.21 | 0/3 |
| PTV_D95 | AS vs DS | -0.47 | -1.01 | 1.49 | +0.01 ± 1.31 | 2/3 |
|  | BS vs DS | -0.64 | -0.87 | -0.19 | -0.57 ± 0.35 | **3/3** |
|  | CS vs DS | -0.06 | -0.16 | 0.54 | +0.10 ± 0.38 | 2/3 |
|  | PS vs DS | 0.20 | -0.41 | 1.43 | +0.40 ± 0.94 | 1/3 |
| PTV_mean | AS vs DS | -0.34 | -0.86 | 1.18 | -0.01 ± 1.06 | 2/3 |
|  | BS vs DS | -0.50 | -0.67 | -0.73 | -0.64 ± 0.12 | **3/3** |
|  | CS vs DS | -0.32 | -0.12 | 0.03 | -0.14 ± 0.18 | 2/3 |
|  | PS vs DS | 0.23 | -0.34 | 1.23 | +0.37 ± 0.79 | 1/3 |
| PTV_CI | AS vs DS | 0.04 | -0.01 | 0.06 | +0.03 ± 0.03 | 1/3 |
|  | BS vs DS | 0.05 | 0.00 | -0.14 | -0.03 ± 0.10 | 2/3 |
|  | CS vs DS | 0.01 | 0.00 | -0.03 | -0.01 ± 0.02 | 2/3 |
|  | PS vs DS | 0.04 | 0.00 | 0.07 | +0.04 ± 0.03 | 1/3 |
| PTV_HI | AS vs DS | -0.24 | -0.70 | 1.61 | +0.22 ± 1.22 | 2/3 |
|  | BS vs DS | -0.41 | -0.86 | 2.98 | +0.57 ± 2.10 | 2/3 |
|  | CS vs DS | 1.76 | -0.16 | 3.06 | +1.55 ± 1.62 | 1/3 |
|  | PS vs DS | 0.05 | -0.20 | 1.12 | +0.32 ± 0.70 | 1/3 |
| Body_mean | AS vs DS | 0.01 | -0.18 | 0.35 | +0.06 ± 0.27 | 1/3 |
|  | BS vs DS | -0.03 | -0.12 | -0.05 | -0.07 ± 0.05 | **3/3** |
|  | CS vs DS | -0.03 | 0.00 | 0.08 | +0.02 ± 0.06 | 1/3 |
|  | PS vs DS | 0.06 | -0.03 | 0.33 | +0.12 ± 0.19 | 1/3 |
| Lung_L_v5 | AS vs DS | -0.01 | -0.01 | 0.00 | -0.00 ± 0.01 | 2/3 |
|  | BS vs DS | -0.01 | 0.00 | -0.01 | -0.01 ± 0.00 | **3/3** |
|  | CS vs DS | 0.01 | 0.00 | -0.01 | +0.00 ± 0.01 | 1/3 |
|  | PS vs DS | 0.00 | 0.00 | 0.01 | +0.00 ± 0.01 | 0/3 |
| Lung_L_v20 | AS vs DS | -0.01 | -0.02 | 0.00 | -0.01 ± 0.01 | 2/3 |
|  | BS vs DS | -0.01 | -0.01 | -0.02 | -0.01 ± 0.01 | **3/3** |
|  | CS vs DS | 0.00 | 0.00 | 0.01 | +0.00 ± 0.01 | 2/3 |
|  | PS vs DS | 0.00 | -0.01 | 0.01 | +0.00 ± 0.01 | 1/3 |
| Lung_L_mean | AS vs DS | -0.50 | -1.20 | 0.83 | -0.29 ± 1.03 | 2/3 |
|  | BS vs DS | -0.45 | -0.67 | -1.12 | -0.75 ± 0.34 | **3/3** |
|  | CS vs DS | -0.16 | -0.14 | 0.24 | -0.02 ± 0.22 | 2/3 |
|  | PS vs DS | -0.05 | -0.31 | 1.07 | +0.24 ± 0.73 | 2/3 |
| Lung_R_v5 | AS vs DS | 0.01 | 0.00 | 0.00 | +0.00 ± 0.00 | 1/3 |
|  | BS vs DS | 0.00 | 0.00 | -0.01 | -0.00 ± 0.01 | 1/3 |
|  | CS vs DS | 0.00 | 0.00 | 0.00 | -0.00 ± 0.00 | 1/3 |
|  | PS vs DS | 0.00 | 0.00 | 0.00 | -0.00 ± 0.00 | 2/3 |

**Table S19.** Seed-specific paired differences in prediction error between each auxiliary-supervision configuration and the corresponding dose-only baseline (DS) under the composite-loss setting. For each random seed $s$(42, 123, and 456), $\Delta_{s}$denotes the difference in absolute ground-truth (GT) endpoint error between the auxiliary-supervision model and DS, calculated on the same held-out test plans. Negative $\Delta_{s}$values indicate lower absolute error than DS (improvement), whereas positive values indicate higher error. Mean $\Delta\pm\mathrm{SD}_{\Delta}$ summarizes the mean and sample standard deviation of the three seed-specific $\Delta_{s}$values. “Seeds improved” indicates the number of random seeds for which $\Delta_{s}<0$. Values are rounded to two decimal places for presentation; the classification of improved seeds is based on the full-precision values before rounding. Abbreviations: AS, all auxiliary tasks; BS, beam supervision; CS, center supervision; PS, patient-structure supervision; DS, dose-only baseline; MAE, mean absolute error; RMSE, root mean squared error; PTV, planning target volume; CI, conformity index; HI, homogeneity index.

**Table S20. Seed-specific paired endpoint effects relative to DS under MSE-only training**

| **Metric** | **Comparison** | **Δ42** | **Δ123** | **Δ456** | **Mean Δ ± SDΔ** | **Seeds improved** |
| --- | --- | --- | --- | --- | --- | --- |
| MAE | AS vs DS | 0.07 | -0.13 | -0.37 | -0.14 ± 0.22 | 2/3 |
|  | BS vs DS | 0.06 | 0.09 | -0.10 | +0.02 ± 0.10 | 1/3 |
|  | CS vs DS | 0.09 | 0.08 | -0.33 | -0.05 ± 0.24 | 1/3 |
|  | PS vs DS | 0.02 | 0.01 | -0.05 | -0.01 ± 0.04 | 1/3 |
| RMSE | AS vs DS | 0.06 | -0.02 | -0.35 | -0.10 ± 0.22 | 2/3 |
|  | BS vs DS | 0.04 | 0.33 | -0.21 | +0.06 ± 0.27 | 1/3 |
|  | CS vs DS | 0.14 | 0.28 | -0.53 | -0.04 ± 0.43 | 1/3 |
|  | PS vs DS | -0.05 | 0.04 | -0.02 | -0.01 ± 0.05 | 2/3 |
| Cord_max | AS vs DS | -0.45 | -0.67 | 0.66 | -0.16 ± 0.71 | 2/3 |
|  | BS vs DS | 0.44 | 1.90 | 0.48 | +0.94 ± 0.83 | 0/3 |
|  | CS vs DS | 1.69 | 3.28 | -0.65 | +1.44 ± 1.98 | 1/3 |
|  | PS vs DS | 0.31 | -0.12 | 0.66 | +0.28 ± 0.39 | 1/3 |
| Heart_mean | AS vs DS | -0.13 | 0.36 | 0.55 | +0.26 ± 0.35 | 1/3 |
|  | BS vs DS | 0.27 | 0.52 | -0.01 | +0.26 ± 0.26 | 1/3 |
|  | CS vs DS | -0.02 | 0.51 | 0.06 | +0.18 ± 0.28 | 1/3 |
|  | PS vs DS | -0.11 | 0.14 | 0.15 | +0.06 ± 0.15 | 1/3 |
| Heart_v5 | AS vs DS | 0.00 | -0.02 | 0.00 | -0.00 ± 0.01 | 1/3 |
|  | BS vs DS | 0.02 | -0.01 | 0.00 | +0.00 ± 0.02 | 1/3 |
|  | CS vs DS | 0.00 | -0.01 | 0.01 | -0.00 ± 0.01 | 1/3 |
|  | PS vs DS | 0.00 | 0.00 | 0.00 | +0.00 ± 0.01 | 1/3 |
| Breast_R_mean | AS vs DS | 0.47 | -1.21 | -0.67 | -0.47 ± 0.86 | 2/3 |
|  | BS vs DS | 0.27 | -1.41 | 0.14 | -0.33 ± 0.94 | 1/3 |
|  | CS vs DS | -0.20 | -1.39 | -0.52 | -0.71 ± 0.62 | 3/3 |
|  | PS vs DS | 0.45 | 0.72 | -0.24 | +0.31 ± 0.50 | 1/3 |
| PTV_D95 | AS vs DS | -0.20 | 0.45 | -2.93 | -0.89 ± 1.80 | 2/3 |
|  | BS vs DS | 0.30 | 2.54 | -0.83 | +0.67 ± 1.72 | 1/3 |
|  | CS vs DS | 0.87 | 2.24 | -2.60 | +0.17 ± 2.50 | 1/3 |
|  | PS vs DS | -0.08 | -0.31 | 0 | -0.13 ± 0.16 | 2/3 |
| PTV_mean | AS vs DS | -0.41 | 0.40 | -2.46 | -0.82 ± 1.47 | 2/3 |
|  | BS vs DS | -0.06 | 2.30 | 0.08 | +0.77 ± 1.32 | 1/3 |
|  | CS vs DS | 0.49 | 1.96 | -1.88 | +0.19 ± 1.94 | 1/3 |
|  | PS vs DS | -0.01 | -0.3 | 0.01 | -0.10 ± 0.17 | 2/3 |
| PTV_CI | AS vs DS | -0.03 | 0.10 | -0.25 | -0.06 ± 0.18 | 2/3 |
|  | BS vs DS | 0.01 | 0.20 | 0.01 | +0.07 ± 0.11 | 0/3 |
|  | CS vs DS | 0.08 | 0.20 | -0.16 | +0.04 ± 0.18 | 1/3 |
|  | PS vs DS | 0.01 | -0.03 | 0.00 | -0.01 ± 0.02 | 1/3 |
| PTV_HI | AS vs DS | 1.40 | -1.18 | -2.68 | -0.82 ± 2.06 | 2/3 |
|  | BS vs DS | 1.71 | 0.40 | -4.93 | -0.94 ± 3.52 | 1/3 |
|  | CS vs DS | 0.77 | 0.16 | -4.32 | -1.13 ± 2.78 | 1/3 |
|  | PS vs DS | -0.43 | -0.06 | 0.05 | -0.15 ± 0.26 | 2/3 |
| Body_mean | AS vs DS | 0.04 | 0.13 | 0.06 | +0.07 ± 0.05 | 0/3 |
|  | BS vs DS | 0.09 | 0.70 | 0.06 | +0.28 ± 0.36 | 0/3 |
|  | CS vs DS | 0.17 | 0.60 | -0.25 | +0.18 ± 0.42 | 1/3 |
|  | PS vs DS | -0.01 | -0.01 | 0.1 | +0.02 ± 0.07 | 2/3 |
| Lung_L_v5 | AS vs DS | 0.00 | -0.01 | -0.01 | -0.01 ± 0.00 | **3/3** |
|  | BS vs DS | 0.00 | 0.00 | -0.01 | -0.00 ± 0.00 | 2/3 |
|  | CS vs DS | -0.01 | -0.01 | 0.01 | -0.00 ± 0.01 | 2/3 |
|  | PS vs DS | -0.01 | -0.01 | 0.00 | -0.01 ± 0.00 | **3/3** |
| Lung_L_v20 | AS vs DS | 0.00 | 0.00 | 0.01 | +0.00 ± 0.01 | 1/3 |
|  | BS vs DS | 0.00 | 0.03 | 0.01 | +0.01 ± 0.01 | 0/3 |
|  | CS vs DS | 0.02 | 0.03 | 0.00 | +0.02 ± 0.01 | 0/3 |
|  | PS vs DS | 0.00 | 0.00 | 0.00 | +0.00 ± 0.00 | 1/3 |
| Lung_L_mean | AS vs DS | -0.23 | 0.27 | 0.46 | +0.17 ± 0.36 | 1/3 |
|  | BS vs DS | 0.08 | 2.09 | 0.62 | +0.93 ± 1.04 | 0/3 |
|  | CS vs DS | 1.17 | 1.69 | -0.23 | +0.88 ± 1.00 | 1/3 |
|  | PS vs DS | 0.09 | 0.21 | 0.27 | +0.19 ± 0.09 | 0/3 |
| Lung_R_v5 | AS vs DS | 0.00 | 0.00 | 0.00 | +0.00 ± 0.00 | 1/3 |
|  | BS vs DS | 0.00 | 0.00 | 0.00 | -0.00 ± 0.00 | 1/3 |
|  | CS vs DS | 0.00 | 0.00 | 0.00 | +0.00 ± 0.00 | 1/3 |
|  | PS vs DS | 0.00 | 0.00 | 0.00 | +0.00 ± 0.00 | 2/3 |

**Table S20.** Seed-specific paired differences in prediction error between each auxiliary-supervision configuration and the corresponding dose-only baseline (DS) under the MSE-only primary dose objective. For each random seed $s$(42, 123, and 456), $\Delta_{s}$denotes the difference in absolute ground-truth (GT) endpoint error between the auxiliary-supervision model and DS, calculated on the same held-out test plans. Negative $\Delta_{s}$values indicate lower absolute error than DS (improvement), whereas positive values indicate higher error. Mean $\Delta\pm\mathrm{SD}_{\Delta}$summarizes the mean and sample standard deviation of the three seed-specific $\Delta_{s}$values. “Seeds improved” indicates the number of random seeds for which $\Delta_{s}<0$. Values are rounded to two decimal places for presentation; the classification of improved seeds is based on the full-precision values before rounding. Abbreviations: AS, all auxiliary tasks; BS, beam supervision; CS, center supervision; PS, patient-structure supervision; DS, dose-only baseline; MSE, mean squared error; MAE, mean absolute error; RMSE, root mean squared error; PTV, planning target volume; CI, conformity index; HI, homogeneity index.

**Table S21. Comparison of seed-wise paired endpoint effects relative to DS under composite-loss and MSE-only training**

| **Metric** | **Comparison** | **Composite loss Mean Δ ± SDΔ** | **Seeds improved** | **MSE-only Mean Δ ± SDΔ** | **Seeds improved** | **Change in seeds improved** |
| --- | --- | --- | --- | --- | --- | --- |
| **MAE (Gy)** | AS vs DS | +0.08 ± 0.23 | 1/3 | −0.14 ± 0.22 | 2/3 | increased |
|  | BS vs DS | +0.01 ± 0.11 | 1/3 | +0.02 ± 0.10 | 1/3 | unchanged |
|  | CS vs DS | +0.06 ± 0.09 | 1/3 | −0.05 ± 0.24 | 1/3 | unchanged |
|  | PS vs DS | +0.10 ± 0.19 | 1/3 | −0.01 ± 0.04 | 1/3 | unchanged |
| **RMSE (Gy)** | AS vs DS | +0.11 ± 0.41 | 1/3 | −0.10 ± 0.22 | 2/3 | increased |
|  | BS vs DS | +0.00 ± 0.20 | 1/3 | +0.06 ± 0.27 | 1/3 | unchanged |
|  | CS vs DS | +0.12 ± 0.16 | 1/3 | −0.04 ± 0.43 | 1/3 | unchanged |
|  | PS vs DS | +0.16 ± 0.31 | 1/3 | −0.01 ± 0.05 | 2/3 | increased |
| **PTV D95 (Gy)** | AS vs DS | +0.01 ± 1.31 | 2/3 | −0.89 ± 1.80 | 2/3 | unchanged |
|  | BS vs DS | −0.57 ± 0.35 | 3/3 | +0.67 ± 1.72 | 1/3 | decreased |
|  | CS vs DS | +0.10 ± 0.38 | 2/3 | +0.17 ± 2.50 | 1/3 | decreased |
|  | PS vs DS | +0.40 ± 0.94 | 1/3 | −0.13 ± 0.16 | 2/3 | increased |
| **PTV mean (Gy)** | AS vs DS | −0.01 ± 1.06 | 2/3 | −0.82 ± 1.47 | 2/3 | unchanged |
|  | BS vs DS | −0.64 ± 0.12 | 3/3 | +0.77 ± 1.32 | 1/3 | decreased |
|  | CS vs DS | −0.14 ± 0.18 | 2/3 | +0.19 ± 1.94 | 1/3 | decreased |
|  | PS vs DS | +0.37 ± 0.79 | 1/3 | −0.10 ± 0.17 | 2/3 | increased |
| **Cord max (Gy)** | AS vs DS | −0.30 ± 0.60 | 2/3 | −0.16 ± 0.71 | 2/3 | unchanged |
|  | BS vs DS | −0.56 ± 0.13 | 3/3 | +0.94 ± 0.83 | 0/3 | decreased |
|  | CS vs DS | −0.10 ± 0.37 | 2/3 | +1.44 ± 1.98 | 1/3 | decreased |
|  | PS vs DS | +0.33 ± 0.33 | 1/3 | +0.28 ± 0.39 | 1/3 | unchanged |
| **LungL V20** | AS vs DS | −0.01 ± 0.01 | 2/3 | +0.00 ± 0.01 | 1/3 | decreased |
|  | BS vs DS | −0.01 ± 0.01 | 3/3 | +0.01 ± 0.01 | 0/3 | decreased |
|  | CS vs DS | +0.00 ± 0.01 | 2/3 | +0.02 ± 0.01 | 0/3 | decreased |
|  | PS vs DS | +0.00 ± 0.01 | 1/3 | +0.00 ± 0.00 | 1/3 | unchanged |
| **Heart Dmean (Gy)** | AS vs DS | −0.16 ± 0.40 | 1/3 | +0.26 ± 0.35 | 1/3 | unchanged |
|  | BS vs DS | −0.27 ± 0.46 | 2/3 | +0.26 ± 0.26 | 1/3 | decreased |
|  | CS vs DS | +0.16 ± 0.25 | 1/3 | +0.18 ± 0.28 | 1/3 | unchanged |
|  | PS vs DS | −0.03 ± 0.15 | 1/3 | +0.06 ± 0.15 | 1/3 | unchanged |

**Table S21.** Seed-wise paired differences in absolute ground-truth (GT) endpoint error between each auxiliary-supervision configuration and the dose-only baseline (DS) under the composite-loss and MSE-only settings. For each random seed, Δ was defined as the absolute endpoint error of the auxiliary-supervision model minus that of the corresponding DS model. Thus, Δ < 0 indicates lower absolute GT error than DS (improvement), whereas Δ > 0 indicates higher error. Mean Δ ± SDΔ summarizes the mean and sample standard deviation of the three seed-specific paired differences. “Seeds improved” denotes the number of random seeds showing lower absolute endpoint error than DS. “Change in seeds improved” indicates whether the number of improved seeds increased, decreased, or remained unchanged when changing from composite-loss to MSE-only training. Values are rounded to two decimal places for presentation; seed-improvement classification is based on the full-precision values before rounding.

Optimization coupling was further assessed by measuring the gradient contribution of each weighted auxiliary loss to the dose-prediction network. Relative gradient magnitude was defined as the gradient norm of the weighted auxiliary loss with respect to the DoseNet parameters, normalized by the corresponding dose-loss gradient norm. Seed-specific gradient statistics under the composite-loss and MSE-only settings are reported in Tables S22 and S23, respectively. The composite-loss gradient table is currently Table S22, whereas the MSE-only gradient table is Table S23.

To examine whether the Beam and Center auxiliary tasks used information contained in the predicted dose beyond that available from CT alone, additional matched auxiliary-input experiments were performed. For each random seed, the corresponding pretrained DS model was frozen and used to generate dose predictions. Beam and Center networks were then trained under matched CT + predicted-dose and CT-only conditions, with the latter implemented using a zero-valued second input channel to preserve the same network architecture. Initialization, data order, and other training settings were matched between the two input conditions. Inclusion of the predicted-dose input reduced Beam-task loss across all three seeds, whereas no consistent improvement was observed for the Center task. These analyses therefore distinguish direct optimization coupling from dependence of the auxiliary task on predicted-dose information. The original appendix already describes this matched-input design and its Beam-versus-Center result in this form.

**Table S22. Composite-loss task-specific relative gradient contributions**

| **Model** | **Auxiliary task** | **Seed** | **Median relative gradient** | **IQR** | **Cross-seed mean ± SD** | **Connected** |
| --- | --- | --- | --- | --- | --- | --- |
| AS | Beam | 42 | 0.46% | 0.242–0.935% | 0.296 ± 0.150% | 124/124 |
|  | Beam | 123 | 0.26% | 0.137–0.743% |  | 124/124 |
|  | Beam | 456 | 0.17% | 0.0647–0.731% |  | 124/124 |
|  | Center | 42 | 0.00158% | 0.000342–0.002668% | 0.00134 ± 0.00030% | 124/124 |
|  | Center | 123 | 0.00143% | 0.000590–0.002397% |  | 124/124 |
|  | Center | 456 | 0.00101% | 0.000154–0.00242% |  | 124/124 |
|  | Patient | 42 | 0% | 0–0% | 0 ± 0% | 0/124 |
|  | Patient | 123 | 0% | 0–0% |  | 0/124 |
|  | Patient | 456 | 0% | 0–0% |  | 0/124 |
| BS | Beam | 42 | 0.52% | 0.218–1.032% | 0.352 ± 0.142% | 124/124 |
|  | Beam | 123 | 0.26% | 0.0914–0.680% |  | 124/124 |
|  | Beam | 456 | 0.28% | 0.0485–0.514% |  | 124/124 |
| CS | Center | 42 | 0.00109% | 0.000263–0.001630% | 0.00116 ± 0.00023% | 124/124 |
|  | Center | 123 | 0.00142% | 0.000847–0.002379% |  | 124/124 |
|  | Center | 456 | 0.00098% | 0.000413–0.002087% |  | 124/124 |

**Table S22.** Relative gradient was defined as the gradient norm of the weighted auxiliary loss with respect to the dose-prediction network parameters, normalized by the corresponding dose-loss gradient norm. Median and IQR are reported for each seed. Cross-seed summaries are calculated from the three seed-specific median relative gradients. “Connected” indicates the number of sampled checkpoints with a non-zero auxiliary gradient to the dose-prediction network.

**Table S23. MSE-only task-specific relative gradient contributions**

| **Model** | **Auxiliary task** | **Seed** | **Median relative gradient** | **IQR** | **Cross-seed mean ± SD** |
| --- | --- | --- | --- | --- | --- |
| AS | Beam | 42 | 0.854% | 0.529–1.336% | 0.788 ± 0.057% |
|  |  | 123 | 0.752% | 0.451–1.458% |  |
|  |  | 456 | 0.759% | 0.382–1.466% |  |
|  | Center | 42 | 0.0736% | 0.0584–0.1369% | 0.0748 ± 0.0110% |
|  |  | 123 | 0.0644% | 0.0412–0.0929% |  |
|  |  | 456 | 0.0863% | 0.0501–0.1320% |  |
|  | Patient | 42 | 0% | 0–0% | 0 ± 0% |
|  |  | 123 | 0% | 0–0% |  |
|  |  | 456 | 0% | 0–0% |  |
| BS | Beam | 42 | 0.932% | 0.629–1.313% | 0.764 ± 0.163% |
|  |  | 123 | 0.607% | 0.391–1.020% |  |
|  |  | 456 | 0.752% | 0.449–1.123% |  |
| CS | Center | 42 | 0.0988% | 0.0746–0.1309% | 0.0937 ± 0.0060% |
|  |  | 123 | 0.0871% | 0.0550–0.1581% |  |
|  |  | 456 | 0.0951% | 0.0486–0.1655% |  |

**Table S23.** Task-specific relative gradient contributions to the dose-prediction network under the MSE-only primary dose objective. Relative gradient contribution was defined as the gradient norm of the weighted auxiliary loss with respect to the dose-prediction network parameters, normalized by the corresponding gradient norm of the primary MSE dose loss. Median and interquartile range (IQR) are reported across sampled training checkpoints for each random seed. Cross-seed mean ± standard deviation (SD) was calculated from the three seed-specific median relative gradient contributions. A value of 0% for the Patient task indicates the absence of a direct auxiliary-gradient pathway from the Patient loss to the dose-prediction network under the implemented computational graph. Abbreviations: AS, all auxiliary tasks; BS, dose + beam supervision; CS, dose + center supervision; IQR, interquartile range; MSE, mean squared error; SD, standard deviation.

# Supplement F. Additional visualizations

This supplement provides additional qualitative case-based visualizations of predicted dose distributions, voxel-wise errors, and DVH profiles. These figures are intended as descriptive support for the cohort-level quantitative analyses and are not used as primary evidence for model comparison.

## F.1 Visualization protocol and case selection

All visualizations follow a consistent protocol. For each case, we present: (a) axial dose maps,(b) voxel-wise difference maps (Prediction − GT), and (c) dose–volume histogram (DVH) comparisons for the ground truth (GT) and the DS, BS, and AS configurations.

All panels use identical color scales, display windows, and plotting settings to facilitate direct visual comparison across task configurations and across cases. To avoid post hoc selection of visually favorable examples, test cases were randomly sampled from the top 15% of the test set ranked by DS performance (based on plan-level voxel-wise MAE). These case-level visualizations are descriptive and are not intended to replace cohort-level comparisons.

## F.2 Representative case visualization

**Figure S3 Additional case-based visualization of dose prediction.** Dose maps, voxel-wise difference maps (Prediction − GT), and DVH comparisons are shown for a representative test case comparing GT with DS, BS, and AS. This figure provides a qualitative view of spatial dose agreement and localized discrepancies.

## F.3 Additional representative cases from the high-performing DS subset

Figures S4–S5 provide two additional examples following the same visualization format as Fig. S3. Across these cases, all configurations reproduce the overall dose distribution patterns, while visually noticeable discrepancies remain predominantly localized near the PTV boundary and high-dose gradient transition regions. These observations are consistent with the representative example in the main text.

**Figure S4 Additional case-based visualization of dose prediction (Case 1).** Dose maps (a), voxel-wise difference maps (Prediction − GT) (b), and DVH comparisons (c) are shown for an additional test case, comparing GT with DS, BS, and AS. Visualization settings, including dose color scales and difference-map windows, are identical to those used in Fig. S3. The figure provides an additional qualitative example of model agreement and localized discrepancies, particularly near the PTV boundary and high-dose gradient regions.

**Figure S5 Additional case-based visualization of dose prediction (Case 2).** Dose maps (a), voxel-wise difference maps (Prediction − GT) (b), and DVH comparisons (c) are shown for a second additional test case, comparing GT with DS, BS, and AS using the same visualization settings as Fig. S3. This example further illustrates the spatial patterns of agreement and localized discrepancies described in the main text and complements the representative case shown in Fig. S3.

# Supplement G. Evaluation Metrics & Statistical Analysis

Evaluation metrics and statistical procedures used in this study are defined in the following sections. Section G.1 introduces voxel-wise aggregated error metrics, Section G.2 defines the spatial overlap metric, Sections G.3–G.4 describe DVH-based endpoints and target-specific quality indices, and Section G.5 details the statistical tests and reporting conventions used for model comparisons. For each plan, all metrics were computed separately for each random seed and then averaged across three seeds to obtain plan-level values; cohort-level results were subsequently summarized across the 650 held-out test plans using these plan-level values.

## G.1 Voxel-wise aggregated dose evaluation

Voxel-wise aggregated error metrics were used to quantify voxel-level agreement between the predicted dose distribution and the ground truth. All dose values were expressed in Gy and evaluated within the patient body region only (voxels inside the external body contour; background voxels outside the body were excluded from all computations). Let $D_{\text{pred},i}$ and $D_{\text{true},i}$ denote the predicted and ground-truth dose of the $i$-th voxel, respectively, where $i\in\Omega_{\text{body}}$ and $N=\mid\Omega_{\text{body}}\mid$ is the number of voxels within the body region.

**(a) Root Mean Squared Error (RMSE)**

$RMSE=\sqrt{\frac{1}{N}\sum_{i=1}^{N} \left( D_{pred,i} - D_{true,i} \right)^{2}}$ (25)

RMSE quantifies the average deviation between the predicted and ground-truth dose distributions, with smaller values indicating higher prediction accuracy.

**(b) Mean Absolute Error (MAE)**

$MAE=\frac{1}{N}\sum_{i=1}^{N} \mid D_{pred,i}-D_{true,i}\mid$ (26)

MAE summarizes the average absolute voxel-wise deviation and is less sensitive to isolated large errors than RMSE.

## G.2 Spatial overlap metric for isodose regions

**(a) Dice Similarity Coefficient (DSC)**

Spatial agreement between predicted and ground-truth dose distributions was evaluated using the Dice Similarity Coefficient (DSC) computed on thresholded isodose regions. For a given dose threshold $T$(Gy), the isodose region was defined as the set of voxels receiving at least $T$Gy, i.e., $\left\{ v\mid D(v)\geq T \right\}$. When an ROI mask was specified, the region was further restricted by set intersection, i.e., $\{v\mid D(v)\geq T\}\cap\text{ROI}$. In this study, DSC was computed across thresholds $T=\left\{ 5,10,20,30,40,50 \right\}$ Gy both on the Body and the PTV regions. Because thresholds start at 5 Gy, voxels outside the patient body typically do not contribute to the isodose regions.

For each threshold $T$, DSC was computed as

$DSC(T)=\frac{2\mid S_{\text{pred}}(T)\cap S_{\text{true}}(T)\mid}{\mid S_{\text{pred}}(T)\mid+\mid S_{\text{true}}(T)\mid}$ (27)

where $S_{\text{pred}}(T)$and $S_{\text{true}}(T)$denote the predicted and ground-truth isodose voxel sets at threshold $T$, and $\mid\cdot\mid$denotes the number of voxels in the set. DSC ranges from 0 to 1, with higher values indicating greater spatial overlap between predicted and reference isodose regions.

## G.3 Dose–Volume Histogram (DVH)–Based Metrics

DVH-based endpoints were computed from voxel-wise dose values within each ground-truth structure mask. For a given structure with voxel set $\Omega_{s}$ and voxel-wise dose values $\left\{ D_{i}{\}}_{i\in\Omega_{s}} \right.$, dose values were extracted and sorted in ascending order. Unless otherwise stated, all DVH endpoints were reported in physical units (Gy). If a structure mask was empty for a given case, DVH metrics for that structure were recorded as missing values and were excluded from cohort-level averaging.

**(a) Dose to** $\boldsymbol{x}\boldsymbol{\%}$ **of the volume (**$\boldsymbol{D}_{\boldsymbol{x}}$**)**

$D_{x}=\text{interp}\left( \{D_{i}{\}}_{i\in\Omega_{s}},\text{ }x\% \right)$ (28)

$D_{x}$is defined as the dose such that $x\%$of the structure volume receives at least $D_{x}$. In implementation, $D_{x}$was obtained by linear interpolation over the sorted voxel-dose distribution within the structure. Typical values used in this study include $x=5$ and $x=95$.

**(b) Volume receiving at least** $\boldsymbol{x}$ **Gy (**$\boldsymbol{V}_{\boldsymbol{x}}$**)**

$V_{x}=\frac{1}{N_{s}}\sum_{i\in\Omega_{s}} \mathbb{I(}D_{i}\geq x)$ (29)

where $N_{s}=\mid\Omega_{s}\mid$ is the number of voxels in the structure and $\mathbb{I(\cdot)}$ is the indicator function. $V_{x}$represents the fraction (0–1) of structure voxels receiving a dose equal to or greater than $x$Gy. In this study, $V_{x}$ was evaluated at clinically relevant thresholds (e.g., $x=5$Gy and $x=20$Gy) to characterize dose exposure of organs at risk.

**(c) Maximum dose (**$\boldsymbol{D}_{\mathbf{max}}$**)**

$D_{\max}=\underset{i\in\Omega_{s}}{max}D_{i}$ (30)

$D_{\max}$ is the maximum voxel dose within the structure and is used to assess hotspot-sensitive endpoints, particularly for serial organs at risk.

**(d) Mean dose (**$\boldsymbol{D}_{\text{mean}}$**)**

$D_{\text{mean}}=\frac{1}{N_{s}}\sum_{i\in\Omega_{s}} D_{i}$ (31)

$D_{\text{mean}}$ summarizes the average dose delivered to the structure and is commonly used to characterize overall dose exposure (e.g., for organs at risk) and dose levels within targets.

## G.4 Target-Specific Quality Indices

Target-specific quality indices were computed for the PTV using the prescription dose as the reference. Let $\Omega_{\text{PTV}}$ denote the set of voxels within the ground-truth PTV mask and $N_{\text{PTV}}=\mid\Omega_{\text{PTV}}\mid$ its voxel count. The prescription dose is denoted as $D_{\text{ref}}$(Gy), and the 95% prescription isodose threshold is defined as $0.95\times D_{\text{ref}}$.

**(a) Conformity Index (CI)**

Conformity of the prescription-level high-dose region to the PTV was quantified using the Paddick Conformity Index:

$CI=\frac{V_{\text{PTV},95}^{2}}{V_{\text{PTV}}\cdot V_{\text{treated},95}}$ (32)

where $V_{\text{PTV},95}$ denotes the number of PTV voxels receiving at least $0.95\times D_{\text{ref}}$, $V_{\text{PTV}}$ denotes the total number of voxels in the PTV, and $V_{\text{treated},95}$ denotes the number of voxels in the entire dose grid receiving at least $0.95\times D_{\text{ref}}$. Volumes were therefore computed as voxel counts on the dose grid. CI ranges from 0 to 1, with higher values indicating that the 95% prescription isodose region more closely conforms to the target. If $V_{\text{treated},95}=0$, CI was set to 0 to avoid division by zero.

**(b) Homogeneity Index (HI)**

Dose homogeneity within the PTV was evaluated using:

$HI=\frac{D_{5}-D_{95}}{D_{\text{ref}}}\times100$ (33)

where $D_{5}$ and $D_{95}$ are the doses such that 5% and 95% of the PTV volume receive at least those doses, respectively, as computed by linear interpolation over the sorted voxel-dose distribution within the PTV (as defined in G.3). HI is reported as a percentage; lower values indicate a more homogeneous dose distribution within the target.

## G.5 Statistical Analysis

All tests were two-sided with a significance threshold of $p<0.05$ unless otherwise stated.

**Paired model comparisons (voxel-wise MAE/RMSE).**

For plan-level voxel-wise MAE and RMSE, each auxiliary-supervised joint optimization configuration (BS, CS, PS, AS) was compared against the baseline DS using Wilcoxon signed-rank tests on paired plan-level values (Supplement E, Table S8). In addition to p-values, mean relative differences (%) with 95% confidence intervals, Cohen’s d with 95% confidence intervals (computed on paired differences), and the proportion of plans with lower error than DS were reported to summarize the direction and magnitude of changes.

**Structure-level DVH bias.**

For DVH-based clinical endpoints, bias was defined as the plan-level difference between the predicted and ground-truth metric values (Prediction − GT). Bias values were summarized as mean with 95% confidence intervals for each model (Supplement E, Table S9-S12) and were interpreted descriptively.

Spatial overlap of isodose regions.

For isodose-region overlap, DSC values were computed at multiple dose thresholds (5–50 Gy) within the Body and PTV regions and summarized as mean ± SD across plans (Supplement E, Table S13-14).

**Sub-task loss stratification.**

For quartile-based analyses, plans in the lowest-loss quartile (Q1) and highest-loss quartile (Q4) of each sub-task loss were compared using two-sided Mann–Whitney U tests, with differences reported as $\Delta=Q4-Q1$(Supplement E, Table S16). These analyses were treated as exploratory and were not interpreted as establishing causal relationships.

**Dataset split balance.**

To assess whether training/validation/test splits exhibited large systematic differences in selected anatomical volumes and clinical plan metrics, two-sided Mann–Whitney U tests were used, and effect sizes ($r$) were reported alongside p-values (Supplement D).

P-values are reported alongside effect sizes and confidence intervals for all analyses. Given the number of endpoints and dose thresholds evaluated, findings were interpreted conservatively rather than relying on statistical significance alone.
